# Panel-based assessment of ecosystem condition as a platform for adaptive and knowledge driven management

**DOI:** 10.1007/s00267-024-02042-9

**Published:** 2024-09-13

**Authors:** Jane U. Jepsen, Per Arneberg, Rolf A. Ims, Anna Siwertsson, Nigel G. Yoccoz, Per Fauchald, Åshild Ø. Pedersen, Gro I. van der Meeren, Cecilie H. von Quillfeldt

**Affiliations:** 1 Norwegian Institute for Nature Research, Department of Arctic Ecology, Fram Centre, 9296 Tromsø, Norway; 2 Institute of Marine Research, Department of Ecosystem Processes, Fram Centre, 9296 Tromsø, Norway; 3 UiT The Arctic University of Norway, Department of Arctic and Marine Biology, 9037 Tromsø, Norway; 4 Norwegian Polar Institute, Fram Centre, 9296 Tromsø, Norway; 5 Institute of Marine Research, Department of Ecosystem Processes, 5392 Storebø, Norway; 6 Present Address: Akvaplan-niva, Fram Centre, 9296 Tromsø, Norway

**Keywords:** Adaptive monitoring and management, Uncertainty, Ecosystem-based management, Ecosystem characteristics, Ecosystem state, Trajectories

## Abstract

Ecosystems are subjected to increasing exposure to multiple anthropogenic drivers. This has led to the development of national and international accounting systems describing the condition of ecosystems, often based on few, highly aggregated indicators. Such accounting systems would benefit from a stronger theoretical and empirical underpinning of ecosystem dynamics. Operational tools for ecosystem management require understanding of natural ecosystem dynamics, consideration of uncertainty at all levels, means for quantifying driver-response relationships behind observed and anticipated future trajectories of change, and an efficient and transparent synthesis to inform knowledge-driven decision processes. There is hence a gap between highly aggregated indicator-based accounting tools and the need for explicit understanding and assessment of the links between multiple drivers and ecosystem condition as a foundation for informed and adaptive ecosystem management. We describe here an approach termed PAEC (Panel-based Assessment of Ecosystem Condition) for combining quantitative and qualitative elements of evidence and uncertainties into an integrated assessment of ecosystem condition at spatial scales relevant to management and monitoring. The PAEC protocol is founded on explicit predictions, termed phenomena, of how components of ecosystem structure and functions are changing as a result of acting drivers. The protocol tests these predictions with observations and combines these tests to assess the change in the condition of the ecosystem as a whole. PAEC includes explicit, quantitative or qualitative, assessments of uncertainty at different levels and integrates these in the final assessment. As proofs-of-concept we summarize the application of the PAEC protocol to a marine and a terrestrial ecosystem in Norway.

## Introduction

Ecosystems are dynamic, and even under stable environmental conditions they will show variability in structure and functions at different spatial and temporal scales (Chapin et al. 2002). Humans have also modified ecosystems over thousands of years, for example by removing megaherbivores and top predators, with large cascading impacts on other ecosystem components (Estes et al. 2011; Barnosky et al. 2016). However, multiple pressures, such as land use, harvesting and climate change have greatly increased in recent decades, causing some ecosystems to leave their past range of variation, resulting in novel ecosystem states (Hobbs et al. 2013). Understanding how such ecosystems may be restored and managed sustainably is challenging and implies that we cannot use a single reference state as a target, but should rather focus on understanding current dynamics and expected change trajectories (Jackson and Hobbs 2009; Beechie et al. 2010; Gann et al. 2019; Williams et al. 2021).

To assess ecosystem changes, and their drivers, and to identify management policies, a number of national and international reporting systems have been developed and implemented in many parts of the world (Maes et al. 2018; Keith et al. 2019; Watson et al. 2020; Edens et al. 2022). These ecosystem accounting systems rely on structured sets of indicators and targets, the latter in terms of distance to different baselines (Lange et al. 2022; Maes et al. 2023). These indicators and baselines share similarities with assessments of the climate system (e.g., by the Intergovernmental Panel on Climate Change (IPCC) or the World Meteorological Organisation). Global climate indicators (Trewin et al. 2021) are, however, derived from a thorough understanding of Earth climate dynamics and of the underlying causes of changes – i.e. they are “scientifically robust” and “covering” the system (Trewin et al. 2021). Similarly, the baseline “pre-industrial period” (1850–1900) used to assess current climate change and political targets (e.g., +1.5° and 2 °C) corresponds to a period where the human influence on climate through the emission of greenhouse gases is considered minimal based on model (Allen et al. 2018) simulations. Data-based baselines are not as clearly defined for ecosystem accounting systems and their associated indicators, as they are not anchored in underlying quantitative or conceptual models of ecosystem dynamics (Bateman and Mace 2020). Indeed, the original attempts by the first ecosystem researchers to base evaluations of ecosystems states and dynamics on well-founded hypotheses (e.g., Odum 1980 for a historical perspective with regard to marsh estuaries ecosystems), appear to have subsided in recent assessments, despite the very large developments in our understanding of ecosystem dynamics (e.g., Barbier and Loreau 2019). This reflects the need to develop an international accounting system which may come at the expense of local relevance, just as national Gross Domestic Product (GDP) cannot be used as a tool to manage local economies (OECD 2019). As for other attempts at “governance by numbers” (Supiot 2017), the links between our knowledge and the accounting indices have become tenuous, and while such international accounts may inform us about the symptoms, the links to potential causes and therefore management policies require a complementary framework.

The lack of adequate data derived from ecosystem-based monitoring designs (Lindenmayer and Likens 2010b; Ims and Yoccoz 2017) may also prevent assessment of core ecosystem state variables. Instead, one uses what is easily accessible, supporting the widespread use of indicators that often are surrogates of uncertain validity with regards to understanding ecosystem state and dynamics (e.g., Lindenmayer et al. 2020). The lack of underlying models has been emphasized as one main obstacle for developing effective monitoring (Lindenmayer and Likens 2010a) and restoration (Lindenmayer 2020), and we argue that the same applies to accounting. This is particularly the case if relevance to management is a main objective (Yoccoz et al. 2001), where identifying causes of observed changes is required to identify effective actions (Ruckelshaus et al. 2020).

Ecosystem-based assessment approaches exist that have focused on single drivers, such as fisheries for marine ecosystems (Link et al. 2020), or pollution for coastal and freshwater ecosystems (Hering et al. 2010). To complement this single driver approach, risk-based frameworks exist to assess potential cumulative effects (e.g., Stelzenmuller et al. 2020; Gissi et al. 2021). Such frameworks focus on potential impacts and less on explicit, “mechanistic” links between drivers and ecosystem dynamics. Our goal here is to devise a general approach that includes multiple drivers, their potential interaction, feedbacks, and internal ecosystem dynamics in a systematic and transparent way. We argue that such an approach should integrate (1) the dynamical properties of ecosystems (Hein et al. 2020), (2) include how current states deviate from reference states (Gann et al. 2019; Maes et al. 2021; Nicholson et al. 2021), (3) how different drivers have affected past change (e.g., legacies) and is expected to affect future changes (forecasting) and (4) account for sources of uncertainty in all steps of the assessment. In line with Williams et al. (2021), we focus on trajectories of change as this escapes the problem of precisely defining reference states when knowledge about such states is poor, and because such a focus provides a good foundation for adaptive ecosystem-based management (Bundy et al. 2010; Williams and Johnson 2017). We implement this approach in a system-specific manner, both because ecosystems typically differ in structure, functioning and driver pressures, and because our knowledge varies among systems. For well-studied ecosystems, this knowledge may derive from decades of research and lead to well-supported conclusions, while for less-studied ecosystems, the approach may lead to identifying knowledge gaps that need to be filled.

Instead of beginning with listing available indicators, as is often done in ecosystem accounting approaches, we begin with what we know about the focal ecosystem. To provide structure to the assessment, we use a set of ecosystem characteristics, which capture key aspects of ecosystem structure and function. Central to the assessment is prior knowledge about how state variables, imbedded in these ecosystem characteristics, are affected by external drivers. Based on available data, we then assess which ecosystem changes can be detected and attributed to external drivers or internal dynamics. The empirical evidence base is built stepwise in the hierarchy from separate state variables via the ecosystem characteristics to the whole ecosystem. In each step, we emphasize the empirical and theoretical support for the links between drivers and ecosystem state variables, as well as on the quality and quantity of data to assess these links quantitatively. When assessing scientific evidence and uncertainties we borrow tools and concepts developed under the IPCC (Mastrandrea et al. 2011) and the Intergovernmental Science-Policy Platform on Biodiversity and Ecosystem Services (IPBES 2018), but aim at providing an operational protocol that can be implemented at scales that are also relevant for devising and assessing management actions (Ruckelshaus et al. 2020; Stelzenmuller et al. 2020). We provide proofs-of-concept of the application of such an approach – termed Panel-based Assessment of Ecosystem Condition (PAEC) - in the form of one terrestrial ecosystem (Low Arctic tundra) and one marine ecosystem (Norwegian North Sea). The former is presented in the main text, while the latter is presented in Supplementary Material S2.

## Overview of the PAEC protocol

**Fig. 1:**
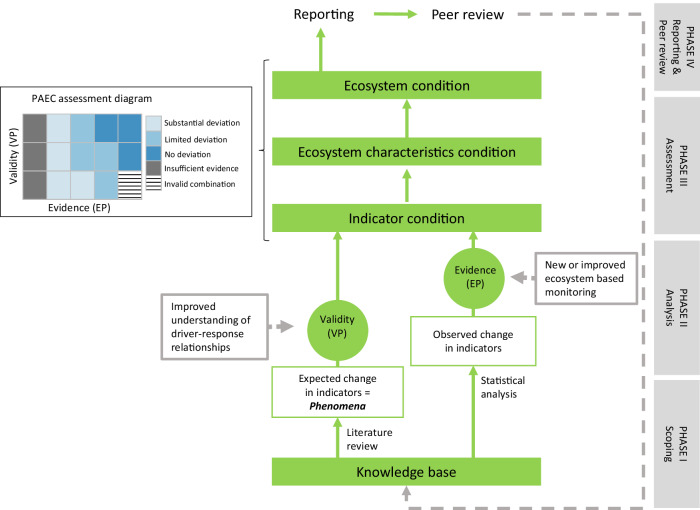
Overview of the four phases (right, gray boxes) in a PAEC assessment, and how they relate to the hierarchical structure of the assessment (green boxes and circles). The assessment builds gradually from an assessment of the knowledge base to assessment of the *Validity* (VP) and *Evidence* (EP) of phenomena associated with individual indicators, to an assessment of the condition of each ecosystem characteristic and the ecosystem as a whole. Avenues for iterative improvement of the quality of assessments (dashed gray arrows) include peer review of the assessment reports, capacity building regarding driver-response relationships resulting in phenomena of higher validity, as well as new or improved data acquisition to strengthen the evidence base. Inset shows the PAEC assessment diagram used to visualize VP and EP assessments of multiple phenomena to facilitate integrated assessments of ecosystem characteristics

PAEC provides a structured protocol for how to combine quantitative and qualitative elements of evidence and uncertainties, into an integrated ecosystem state assessment (Jepsen et al. 2020). The assessments are performed by a scientific panel consisting of members with in-depth knowledge of the focal ecosystem and relevant quantitative methodology. PAEC has a hierarchical structure (Fig. 1) where state variables and derived indicators are nested within a set of ecosystem characteristics that covers major features of ecosystem structure and function. Although there is an important distinction between indicators and state variables, as the former are often surrogates for the latter (cf. Lindenmayer et al. 2015), we will as a matter of convenience use the term indicators in the following. It is possible to define specific ecosystem characteristics for the actual ecosystem, or to use already established hierarchies such as the one used in the United Nations System of Environmental Accounting (UN SEEA; Czúcz et al. 2021; United Nations et al. 2021). In the case of the Norwegian PAEC assessments, the same seven ecosystem characteristics were employed to both case ecosystems. These were: i) *Primary productivity*, 2) *Biomass distribution among trophic levels*, 3) *Functional groups within trophic levels*, 4) *Functionally important species and biophysical structures*, 5) *Landscape-ecological patterns*, 6) *Biological diversity* and 7) *Abiotic factors* (Supplementary Material S1, Supplementary Table S1). A central feature of the PAEC protocol is to use the best available prior knowledge about an ecosystem to predict how ecosystem structure and functions are changing away from a reference condition because of acting external drivers. These predictions are called *phenomena* (Box 1). Following a normal scientific approach, the predictions are tested using observations, and the level of evidence supporting each prediction is assessed. Precisely quantified reference states (e.g., “undisturbed” or “pristine”) are rarely within reach, both scientifically and in terms of estimation and as management objectives. Any assessment of ecosystem condition, however, must necessarily be made relative to a reference state and/or a specified baseline, such as a given year (Gann et al. 2019; Maes et al. 2021). In particular, while a reference state such as “undisturbed” needs to be described, at least in normative terms, it does not need to be quantifiable to be used in a PAEC assessment. Since the most suitable choice of reference and/or baseline will vary depending on the ecosystem in question, and the proposed use of the assessment, PAEC is generic regarding the choice of reference or baseline states.

In Fig. 1 we present an overview of the practical steps (i.e., four phases) in a PAEC assessment and the hierarchy spanning from assessments of the prior knowledge base to the condition of individual indicators, ecosystem characteristics and the ecosystem as a whole. In each of the steps, there is an explicit focus on the different sources of uncertainties related to both theoretical knowledge gaps and lack of data. The uncertainties are addressed quantitatively or qualitatively according to a set of criteria (Supplementary Material S3, Supplementary Table S3). In the following, we describe the content of each of the four phases, as well as how different sources of uncertainties are approached within each phase.

Box 1 Assessment of changes in condition based on phenomena The phenomenon formulated for each indicator serves as a guide throughout the assessment. In most cases phenomena will be expressed qualitatively, e.g., as an expected direction of change in the indicator under the influence of a driver. For instance, *Snow cover duration* (Fig. 3A) is a central indicator for the ecosystem characteristics *Abiotic factors* in Arctic tundra. This indicator is expected to decrease as a result of anthropogenic climate change (Niittynen et al. 2020). The ecological significance of snow cover duration is both related to its maintenance of central functions (e.g., albedo, trophic relationships, phenology, regularity of rodent cycles) and structures (e.g., vegetation layers, snow bed habitats, subnivean space). A strong or consistent decrease in snow cover duration is an adverse development, which over time will jeopardize the continued integrity of an Arctic tundra ecosystem (Callaghan et al. 2011; Ims et al. 2013a). The associated phenomenon is hence formulated as *Shorter season with snow cover*, and this is considered a phenomenon of high validity (as the knowledge of link to the driver and ecosystem consequences from change in indicator values are rated as good). The goals of the subsequent *Analysis phase* (Phase II) and *Assessment phase* (Phase III) are thus to quantify the extent to which this expected development has taken place by estimating the observed rates of change in snow cover duration, and to determine whether the observed changes are sufficiently substantial to be of ecosystem significance, i.e., to determine the level of *evidence for the phenomenon* (EP, See Phase II).

The expected direction of change in an indicator is not always unequivocal, as both increases and decreases in an indicator might be expected dependent on complexity in driver and context. In such cases, the phenomenon might be formulated as a *Change in <indicator*>. A well-documented example of ambiguous expectations to the biotic effects of anthropogenic climate change concerns trends in Arctic vegetation productivity, where both increasing (greening) and decreasing (browning) trends might be expected as a result of anthropogenic climate change interacting with other natural (grazing, browsing) and anthropogenic (land use, herbivore management) drivers (Myers-Smith et al. 2020). Such phenomena might still be of high validity but are more challenging to assess since the level of evidence will often vary depending on the local context (Fig. 3B). Phenomena with low validity can have important roles in assessments by highlighting uncertainties and pointing to key knowledge gaps.

## PHASE I. Scoping

The scoping phase (Fig. 1) begins with defining the conceptual and geographical extent of the ecosystem to be assessed, the reference condition or baseline, and the set of ecosystem characteristics to be used. Poor conceptual or geographical delineation of the ecosystem is a source of both linguistic and epistemic uncertainty in the final assessment (Supplementary Material S3, Supplementary Table S3). This is addressed both in a narrative account of ecosystem delineation, and in the assessment of the spatial representativity of the individual datasets (see below).

System-specific knowledge about structure, processes and drivers will guide the selection of relevant indicators for each ecosystem characteristic. The PAEC protocol does not specify routines for indicator selection, as it might vary greatly from case to case how this is best approached. However, the process of identifying and selecting indicators is helped by the construction of conceptual models of how the ecosystem works (Lindenmayer and Likens 2010b; Ims and Yoccoz 2017). Although our understanding of complex ecosystem processes is most often limited, it is usually possible to depict parts (modules) of the ecosystem in a conceptual model describing the most important ecosystem components and their linkages and pressures. Having such conceptual models will offer guidance for selecting the most relevant indicators for the specific ecosystem, and to start building statistical models used to test the suggested relationships. The structured process of first defining important ecosystem characteristics, and then to use the knowledge about the ecosystem to determine important indicators will contribute to identifying important gaps in e.g., monitoring programs, and thus the possibility to continuously improve the understanding and data availability of the target ecosystem.

**Fig. 2:**
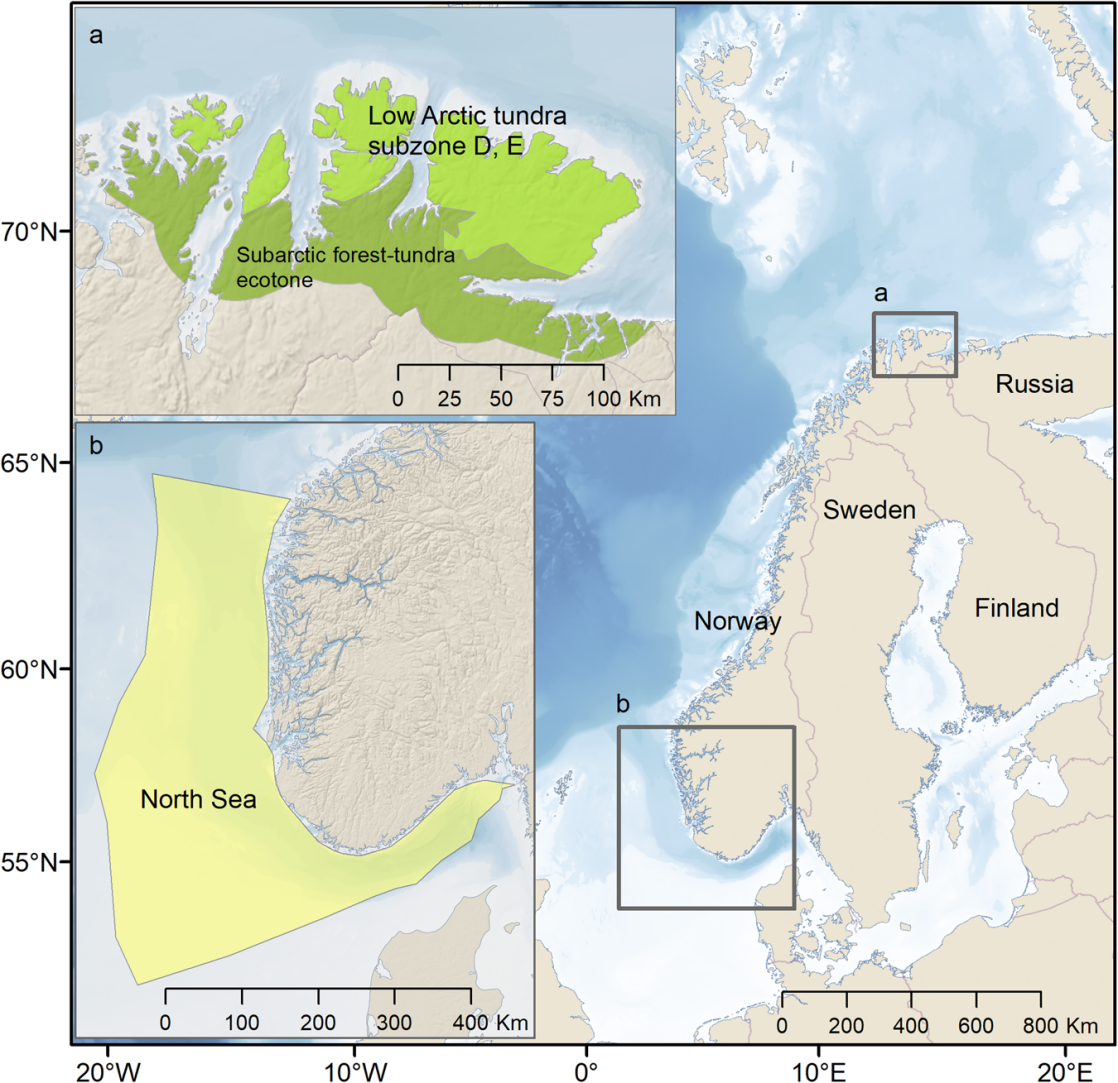
Map of Fennoscandia showing the extent of the assessment areas; the Low Arctic tundra (**a**) and the Norwegian part of the North Sea (**b**). Only the Low Arctic tundra assessment is presented as a case here. The assessment of the Norwegian North Sea is presented as a case in Supplementary Material S2

The formulation of phenomena is a central step in the *Scoping phase*. A phenomenon is a formalized description of how each indicator can be expected to change, based on peer-reviewed literature, as a result of relevant drivers acting on the system (Supplementary Table S3, Box 1). Thus, while phenomena share many features with hypotheses formulated prior to scientific studies, they range in stringency from formally defined quantitative models describing expected cumulative impact of several drivers to, in the case of less information, more speculative expectations of change related to a single driver qualitatively expressed in verbal terms (Box 1). In assessments of ecosystem condition, the focus will most often be on changes in ecosystem condition as a consequence of anthropogenic drivers. In addition to the driver – response relationship, the phenomena also describe the importance of the indicator in the ecosystem (i.e., its ecological significance) by describing how changes in the indicator may affect other linked parts (components or processes) of the ecosystem. The scientific uncertainty of the predictions in the phenomena is assessed in terms of the *Validity of the phenomenon* (VP) based on prior scientific knowledge (Supplementary Table S3, Box 1). Two considerations contribute to the choice of assessment category for VP: 1) the certainty of the link between relevant drivers and changes in the indicator, and 2) the understanding of the importance of change in the indicator for other parts of the ecosystem (Supplementary Table S3, Box 1). The final step in the *Scoping phase* is to identify data sources, which can be used to calculate indicators (Phase II), and to assess the level of evidence for the phenomena (Phase III). Uncertainty brought about by the representativity and accuracy of available data (Supplementary Table S3), is addressed by scoring the temporal and spatial representativity of each dataset for the indicator and phenomena addressed. A PAEC assessment always represents a pre-defined, specific spatial area given by the geographical delineation of the assessment (see Fig. 2 for the delineations of the cases addressed here). The spatial representativity of e.g., data sources are assessed relative to this delineation. The minimum spatial unit is by necessity determined by a combination of the spatial coverage of the underlying data and the spatial scale of underlying ecosystem processes.

## PHASE II. Analysis

In the *Analysis phase* (Fig. 1), observations are used to test the predictions expressed by the phenomena. The assessment of the amount of *evidence for the phenomenon* (EP) includes an evaluation of both statistical evidence (significance) and of ecosystem significance in the case of observed changes (Supplementary Table S3). In some cases (e.g., Henden et al. 2020; Marolla et al. 2021), this assessment can be based on quantitative models of indicator – driver relations. However, for most of the indicators, the statistical analyzes will be based on time series that yields estimates of trajectories of change. Ecosystem significance can be evaluated either by observed changes in other (linked) parts of the assessed ecosystem, or by expected impact on other ecosystem components based on scientific literature. A phenomenon with high level of evidence (EP) is hence one for which we see large or accelerating changes in a direction that is in accordance with the phenomenon prediction, and where the magnitude of these changes is expected or known to be of ecosystem significance.

## PHASE III. Assessment

In the *Assessment phase*, the validity (VP) and evidence (EP) for phenomena are used for graphical representation of the condition of the individual phenomena in the PAEC assessment diagram (Figs. 1 and 4). The visualization also includes the assessment of the knowledge base (data coverage) for each indicator (see Phase I, Supplementary Table S3). The PAEC assessment diagrams visually combine large amounts of information and are an aid to the scientific panel in their assessments of each of the ecosystem characteristics and the ecosystem as a whole.

In a PAEC assessment, conclusions are drawn based on the integration of quantitative and qualitative information, as far as possible retaining all sources of uncertainties (Supplementary Table S3). This permits consideration of interactions and inter-dependency between indicators and between ecosystem characteristics, even if these cannot be explicitly analyzed. The condition of each ecosystem characteristics is scored to one of the three categories: *no, limited*, or *substantial deviation from the reference condition*. This assessment of condition can be challenging, especially when complex ecosystem characteristics must be assessed by many indicators and when associated phenomena are spread across several validity (VP) and evidence (EP) categories in the diagram (see examples of how this may be dealt with in Case section below).

Following the assessment of each ecosystem characteristic, the condition of the whole ecosystem is assessed based on the integration of conditions of all ecosystem characteristics. The assessment is a qualitative, narrative account whose content and structure are detailed by the PAEC protocol. It takes into consideration differences in indicator coverage (Supplementary Table S3), possible interactions between characteristics, and is guided by an understanding of relative importance of the different characteristics for the specific ecosystem.

## PHASE IV. Reporting and review

The PAEC *Reporting phase* (Fig. 1) includes relatively extensive documentation of all the hierarchical steps in the assessment process. In addition to the ecosystem condition assessment, the report also includes chapters on likely future trajectories for the ecosystem and recommendations for monitoring and research. These recommendations are based on the different sources of uncertainty identified during the PAEC process (Supplementary Table S3) and can relate to both insufficient scientific understanding and poor or lack of data (Fig. 1). An independent peer-review of the final assessment report should be prioritized, and resources allocated to consider and include recommendations from this review prior to the next assessment round. For ecosystems subjected to rapid changes, the assessment ought to be repeated frequently (i.e., 5-year intervals). In addition to providing input for management, this will increase confidence in the assessments done and contribute to continuous improvement. To provide consistency between assessment rounds, each new assessment is to be considered an update of the previous assessment, and all deviations between the two should be documented.

## Case study: assessment of ecosystem condition for Norwegian Low Arctic tundra

Application of the PAEC protocol is here illustrated for a terrestrial ecosystem, the Low Arctic tundra in northern Norway. The complete tundra assessment, which additionally includes the High Arctic tundra on the Svalbard Archipelago, can be found in Pedersen et al. (2021). A further case for a marine ecosystem, the Norwegian part of the North Sea, which is described more extensively than the tundra assessment, can be found in Supplementary Material S2, and the complete assessment in Arneberg et al. (2023). The assessments were done as a larger undertaking of assessing ecosystem condition for all marine and terrestrial ecosystem types in Norway, following up a national action plan for biodiversity (Anonymous 2015). They therefore adhere to shared definitions agreed upon within this undertaking, both with respect to the geographical extent of the assessments (Fig. 2), and the reference condition used as a baseline (details below). The first full scale assessment for Norwegian Arctic tundra was conducted by a scientific panel of 21 scientists (Pedersen et al. 2021).

### Delineation of the Low Arctic tundra ecosystem

The Norwegian Low Arctic tundra represents the westernmost fringe of the vast Eurasian tundra biome at 70-71°N, 30°E in NE Norway (Ims et al. 2013b) and belongs to the Low Arctic bio-climatic subzones D and E (Walker et al. 2005). To the north, the tundra borders the ice-free coast of the Barents Sea, while to the south the tundra is gradually transformed – across a tundra-forest ecotone – to northern boreal birch forest (Fig. 2). The present assessment includes the tundra-forest ecotone as it can be expected to influence processes in the tundra (e.g., through mobile organisms; Killengreen et al. 2012) and to be a hotspot for climate change impacts (e.g., emergent insect pest outbreaks; Jepsen et al. (2013)). The dominant land use is reindeer herding, which influence vegetation (e.g., Bråthen et al. 2017) and carnivore communities (Killengreen et al. 2011; Henden et al. 2014). A more detailed description of the ecosystem is provided in (Ims et al. 2013b; Ims et al. 2017).

### Reference condition

The Norwegian *System for assessment of ecological condition* (Nybø and Evju 2017; Anonymous 2023) is an undertaking for assessment of ecosystem condition for all major marine and terrestrial ecosystems in Norway, following up a national action plan for biodiversity (Anonymous 2015). The reference condition, which has been implemented within this system (Nybø and Evju 2017), is “intact ecosystems”. This implies that the fundamental structures, functions, and productivity of the ecosystem are maintained, and that these are not significantly impacted by humans, including anthropogenic climate change. A specific baseline for climate has been chosen as the climate normal period 1961–1990 (Arguez and Vose 2011). This was a relatively cold period compared to the rest of the 20^th^ century (Hanssen-Bauer et al. 2015) and thereby possibly reflecting pre-industrial (*sensu* IPCC; 1850–1900) climatic conditions well. However, we lack data both for the reference condition “intact ecosystems” and for the specific climatic baseline period for many biological indicators, as biological monitoring mostly dates to the early 2000’s and rarely further back than the early 1980’s. Both the Low Arctic tundra and the Norwegian North Sea have been under substantial influence from anthropogenic activities far back in time. The Low Arctic tundra ecosystem has been subjected to harvesting and reindeer herding for centuries, and the region has experienced significant temperature increases since the climatic baseline period. In the North Sea (Supplementary Material S2) industrial fisheries date back to the 19^th^ century (Kerby et al. 2012), and was followed by increasing impacts from nutrient input, offshore industry, and pollution in the early 1900´s. The state of individual indicators and ecosystem characteristics under the reference condition can hence mostly be described in normative terms only without being quantified.

### Individual ecosystem characteristics

Below we summarize the outcome of assessment for three of the seven ecosystem characteristics in Low Arctic tundra, which highlight the most salient aspects of the protocol, as well as for the ecosystem as a whole. For the full assessment, and supporting references, we refer to Pedersen et al. (2021). Supplementary Table S3 specifies and exemplifies how different sources of uncertainty were handled in the different steps of the assessment.

#### Abiotic factors

**Fig. 3:**
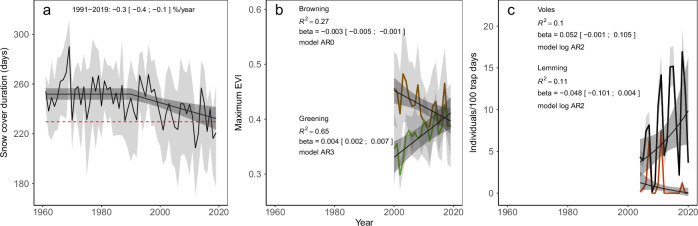
Selected time series of indicators used in the assessment for the ecosystem characteristics *Abiotic factors* (**a**), *Primary productivity* (**b**) and *Functionally important species and biophysical structures* (**c**) for Low-Arctic tundra. Note that time-series of biological indicators are typically shorter than for abiotic factors, and often do not overlap with the chosen baseline for climate (1961–1990). For biological indicators with rapid response to pressures and relatively low natural variation, changes can be assessed even with shorter time-series. Examples of this are the primary productivity indicator (**b**), which typically responds rapidly to changes in climatic conditions. Other biological indicators, such as lemming and vole abundance (**c**), show large natural variation and for these assessments of changes are more uncertain when time-series are short. Details on **a**: The black regression line shows the rate of change (±2SE) in snow cover duration if the indicator value is assumed constant during the climate baseline. Red dashed line indicates the −2SD of the variation observed during the climatic reference period. Details on **b**: brown and green fluctuating lines show the annual mean (±SD) of the maximum EVI for 40 randomly selected pixels within two tundra regions which show overall browning and greening respectively over the observation period. The black regression lines show the rate of change (±2SE). Details on **c**: Black and red fluctuating line show mean (±SE) fall densities of voles and lemmings. The black regression lines show the rate of change (±2SE). To estimate linear rates of change for **b** and **c**, regression models with different structure for the residuals were used. The best fitting model was chosen based on Akaike Information Criterion (AIC). The possible models included in the model selection were: 1) AR0, a standard linear regression with independent residuals, 2) AR1, a 1st order autoregressive model, 3) AR2, a 2nd order autoregressive model, 4) AR3, a 3rd order autoregressive model, 5) ARMA11, a 1st order autoregressive model with a 1st order moving average

**Fig. 4:**
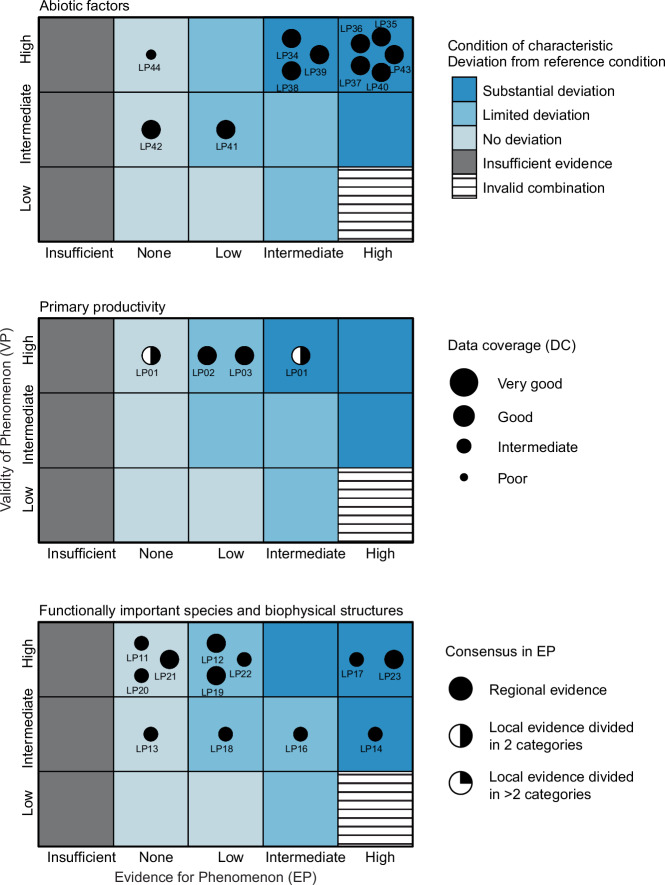
PAEC assessment diagrams for the three ecosystem characteristics exemplified in the case study of Low-Arctic tundra; *Abiotic factors* (top), *Primary productivity* (middle) and *Functionally important species and biophysical structures* (bottom). The diagrams show how all phenomena are placed along the axis of validity (VP) and evidence (EP). Similar diagrams were developed for all seven ecosystem characteristics and used to facilitate the integrated assessment of each ecosystem characteristic and the ecosystem as a whole. Poor data coverage can be accounted for by placing less emphasis on phenomena shown in smaller symbols. Lack of consensus in the level of evidence (EP) for instance due to contrasting trends in different regions or data sources, can be shown by splitting the symbols for EP into half or quarters

**Fig. 5:**
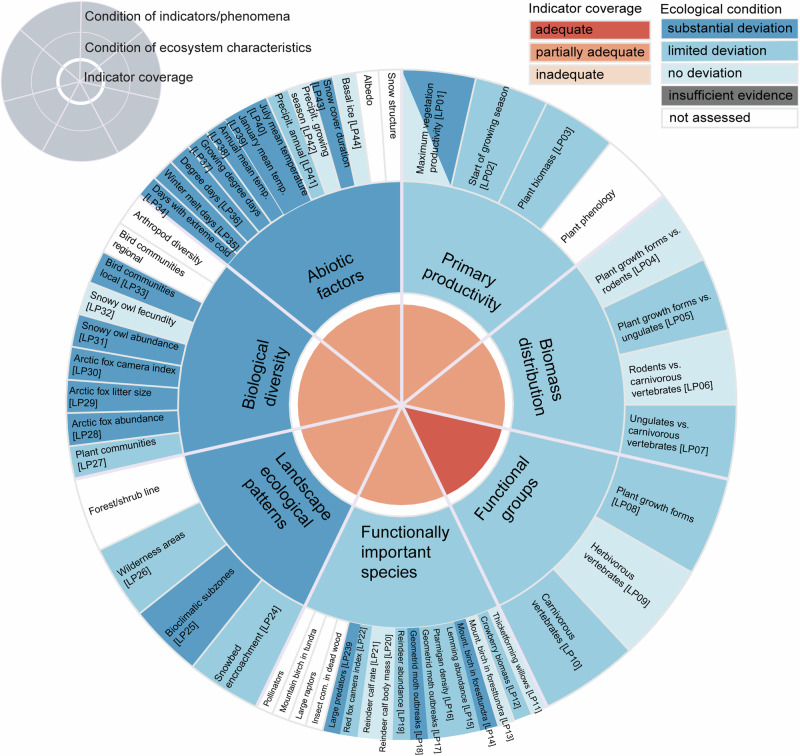
A graphical summary of the assessment of ecological condition for Low-Arctic tundra. The outer ring shows the assessment of condition at the level of individual indicators with associated phenomena IDs in square brackets. Indicators that the scientific panel has recommended for inclusion, but which have not yet been included, are shown in white to illustrate the perceived most important deficiencies in the current indicator set. The middle ring shows the assessment of condition at the level of ecosystem characteristics, and the innermost ring shows the quality of the indicator coverage

This ecosystem characteristic is represented by 11 indicators with 11 associated phenomena of intermediate to high validity (Fig. 4). Overall indicator coverage is assessed as *partially adequate* (Fig. 5). This is due, among others, to the absence of indicators that characterize reflective properties of the surface (albedo) and snow quality, including regional occurrence of rain-on-snow events and subsequent basal icing. The phenomena are derived from previous circumpolar assessments (ACIA 2004; CAFF 2013; AMAP 2017) and are mostly of high validity with well-studied driver-links (higher for phenomena related to temperature than precipitation). The majority (eight of 11) of the phenomena show high evidence for change and are hence located in the category *substantial deviation* (Fig. 4). These are all related to temperature and snow conditions and show changes which can be expected to have profound impact on ecosystem condition over time. This includes a shorter snow season (Fig. 3A), an increasing number of degree days, and a shift away from a historical climatic regime, which have permitted discontinuous permafrost (sub-zero annual mean temperatures). Two of the three phenomena which are in the category *no deviation* or *limited deviation*, are related to precipitation and are considered of less relevance for ecosystem condition than indicators related to temperature and snow/ice. The conclusion of the assessment is hence that this ecosystem characteristic shows *substantial deviation* from the reference condition (Fig. 4).

#### Primary productivity

This ecosystem characteristic is represented by three indicators with three associated phenomena of high validity (Fig. 4), which implies well studied driver-links and a good understanding of the importance of changes in productivity (e.g., Legagneux et al. 2014; Ims et al. 2019) and growing season phenology (e.g., Høye et al. 2007) for the condition of arctic tundra ecosystems. Two indicators relate to growing season attributes (*Maximum vegetation productivity* e.g., “greenness”, and *Start of growing season*), and one relate to plant biomass in selected tundra vegetation types. This is considered a *partially adequate* indicator coverage, mainly due to the absence of field-based indicators of plant phenology. The phenomena show limited evidence for change, with the exception of *Maximum productivity*. The latter shows spatially contrasting evidence for changes (Figs. 3B and 4) that can be attributed to different climate change related mechanisms operating in the tundra (predominantly greening) and the forest-tundra ecotone (greening and browning, the latter due to intensified insect outbreaks), as well as regions which show no trends. For this reason, this phenomenon is divided between two categories (*no deviation* and *substantial deviation*; Fig. 5). The conclusion of the assessment is that this ecosystem characteristic overall shows *limited deviation* from the reference condition (Fig. 5).

#### Functionally important species and biophysical structures

For this ecosystem characteristic, indicators are based on their importance in food webs as resources and consumers or as habitat structures or ecosystem engineers. The ecosystem characteristic is represented by 10 indicators and 13 associated phenomena of intermediate-high validity (Fig. 4). The overall indicator coverage is assessed as *partially adequate* mostly due to a lack of indicators on the key functional groups detritivores and pollinators. Although the phenomena are mostly of high validity and the data coverage of the indicators are either good or very good, the assessment is challenged by a considerable spread of the phenomena on the EP axis (Fig. 4). Three of the phenomena are placed in the category *substantial deviation*. For two of these, this is due to the rapid emergence of climatically intensified outbreaks of insect defoliators that has caused an ecosystem state-shift in the forest-tundra ecotone. The third phenomenon with substantial deviation is the politically decided elimination of large carnivores (in particular wolves) in regions of Norway with reindeer herding. However, the implication of this policy is assessed not to be decisive for the overall assessment of the ecosystem characteristic. An expected dampening of lemming cycles (Ims et al. 2011), due to milder winters with less stable snow cover and higher frequencies of basal ice, is a phenomenon of key importance in the Arctic tundra. This change will have profound impacts on the persistence of Arctic specialist predators. Indications of such dampening are observed (example from one locality in Fig. 3C; note contrast between vole and lemming dynamics). However, short time series, relative to the large natural fluctuations inherent to these populations, means that the level of evidence for this phenomenon is still considered low and the phenomenon (*less frequent, less distinct peaks in the lemming cycle*) is hence located in the category *limited deviation*. Given that the bulk of the phenomena is placed in the *limited deviation* category and that the two phenomena with highest ecological significance is still mostly restricted to the forest-tundra ecotone, the ecosystem characteristic is overall assessed as having *limited deviation* from the reference condition (Fig. 5).

### Overall ecosystem condition

For the tundra ecosystem, the assessment concludes that the ecosystem as whole has *limited deviation* from the reference condition (Fig. 5). The characteristic *Abiotic factors* show *substantial deviation*, owing to a rapid climate change, resulting in the loss, in a climatic sense, of the closely associated bioclimatic tundra subzone D (*Landscape ecological patterns*) from the Norwegian low Arctic. Still, most of the biotic ecosystem characteristics only show *limited deviation* from the reference condition. An exception is *Biological diversity*, which is placed in the category *substantial deviation*, due to substantial declines in some endemic arctic species high in the food chains. Although most of the deviations in the biotic ecosystem characteristics are consistent with phenomena attributed to climate change impacts and change trajectory of biotic state variables towards a borealization of the ecosystem, most of the changes appear to lag behind the documented abiotic change. Exceptions are fast state-transitions in the forest-tundra ecotone associated with pest insect outbreaks that very recently have started to spread to the tundra. Also, biodiversity indicators that are highly linked to changed food web dynamics have displayed fast changes. The mixture of fast and slow changes and the only partially adequate indicator coverage of the biotic ecosystem characteristics renders some uncertainty of the overall assessment.

## Discussion

### A structured approach to ecosystem-level assessments

PAEC is a structured assessment protocol that targets ecosystems at spatial scales that are relevant to ecosystem-based management and monitoring. Thereby, PAEC has a somewhat different purpose than existing national or global ecosystem accounting approaches and arguably remedies some of the problems with these approaches. First, by explicitly focussing on and integrating driver-ecosystem characteristic relationships into the assessment (via phenomena), it allows for a more efficient link to mitigation or adaptation actions, and priorities with regards to what can be managed and with what objectives. Second, by making the assessment specific to ecosystems that are relatively homogeneous in terms of characteristics and effects of drivers, the “pathway to action” (Ruckelshaus et al. 2020) is easier to define. Third, by structuring the analytical process with regards to quality and availability of data, phenomena quantifiable through statistical analyzes, and considerations of all sources of uncertainty in the hierarchical assessment process, it clearly points to where the main knowledge gaps are and how to remedy them.

Applied to Norwegian ecosystems, PAEC uses seven ecosystem characteristics to structure the assessment. Other assessment systems use different structures (e.g., Gann et al. 2019 for ecosystem restoration). Recently the United Nation’s *System of Environmental-Economic Accounting—Ecosystem Accounting* (United Nations et al. 2021) proposed to use 6 ecosystem condition characteristics, grouped in “abiotic”, “biotic” and “landscape” types. While some characteristics are largely identical (e.g., our *Landscape-ecological patterns* versus UN *Landscape and seascape characteristics, describing mosaics of ecosystem types at coarse (landscape, seascape) spatial scales*), others differ in terms of ecosystem emphasis: for example, *primary productivity* defines one ecosystem characteristic in PAEC, whereas it is only one component of a large *Functional state characteristics* in the UN system. Such differences in assessment structure may lead to different conclusions about ecosystem condition, particularly if the structure is the basis for a quantitative weighting scheme as in the UN system. The hierarchical structure of PAEC, with a set of key state variables (indicators) and associated phenomena and their assessed uncertainties at the base, makes the approach transparent and easily amenable to changes in the set of ecosystem characteristics. In particular, choosing different characteristics may be required for highlighting important policy or management targets such as carbon storage or endemic species conservation, but will mainly amount to a reorganization of the PAEC assessment.

Our knowledge of reference states (e.g., “intact” ecosystems in the cases presented here), ecosystem dynamics and drivers of ecosystem changes is often fragmentary. It may therefore seem surprising that some recent accounting systems are aimed at quantitative measures of ecosystem and reference condition on fixed scales (0–1; e.g., Jakobsson et al. 2021; United Nations et al. 2021). No such “scaled” assessment exists for the climate system – the (rounded) 1.5 °C and 2 °C targets are defined in terms of the severity of impacts. The same applies to economic accounting – there is no reference state for economic (in)equality, and no scale measuring how far we are from any “desired” state. While we strongly support a quantification of driver-condition relationships, when our understanding of the system and the underlying data warrant it, it is important to have a protocol that focuses on what is relevant and important for the focal ecosystem, even if only qualitative assessment of the state and changes can be made.

The two ecosystems presented as cases here and in Supplementary Material S2 differ along many axes. Most noticeably they differ with respect to their ecological realms (marine versus terrestrial), the nature and spatio-temporal scale and legacies of human exploitation, the pathways by which they are impacted by recent climate change, as well as their inherent ecological dynamics (food web and landscape structure, biotic interactions, natural fluctuations). It would hence be reasonable to expect that the two scientific panels would encounter quite different challenges in assessing ecosystem condition according to a shared protocol. This proved in fact not to be the case. The two ecosystems are united by the availability of dedicated long-term monitoring programs, which for the tundra system is systematically ecosystem-based and for the North Sea cover a large part of key ecosystem components. As such, they represent unique cases in Norway. The fact that such monitoring programs are in place not only means better availability of data sources, which are more compliant (i.e., combinable) across time and space, but also that the PAEC assessments have been preceded by a decade or more of conceptual and methodological brickwork laying the foundation for ecosystem-level assessments (Beaugrand 2004; Fauchald et al. 2011; Ims et al. 2013b; Ims and Yoccoz 2017; Henden et al. 2020; Ravolainen et al. 2020).

### Attribution of driver-response relationships in a PAEC assessment

A PAEC assessment acknowledges that our ability to attribute driver-response relationships for individual indicators vary along a continuum, where we – at the lowest level – might only be able to make a simple qualitative formulation of the expected relationship between an indicator and its proposed drivers, thus potentially providing a low level of confidence in attribution. At the other end of the continuum are indicators where we can formulate formal statistical models to estimate the strength of often complex causal relationships between multiple drivers and indicator condition, contributing to a higher level of confidence in attribution. PAEC specifies that the understanding of the combined driver-response relationship should be classified in two classes depending on whether this can be considered certain or less certain (e.g., whether they can be attributed with higher or lower confidence). Along with a similar classification of our understanding of the role of the indicator in the ecosystem, this forms the basis for scoring the validity (VP) of PAEC phenomena. A phenomenon of high validity is one where the links to the identified set of drivers are considered relatively certain, and the understanding of the role of the indicator in the ecosystem is considered good. In other words, it represents a scientifically well-founded hypothesis of how anthropogenic drivers are expected to change the condition of an indicator, and the implications such changes may have for the ecosystem being assessed. A low validity on the other hand, is a flag of caution for the assessment panel, meaning that the assessment is made on a less well-founded scientific basis. This will allow the panel to recommend steps to improve the validity of specific phenomena, and through that increase confidence in future assessments. The most common cause of low validity for phenomena is that most ecological response variables are simultaneously subjected to multiple, often interacting, drivers of change. Disentangling these through quantitative modeling of ecological responses as functions of multi-driver impacts, is not an integrated part of the PAEC protocol. We advocate however, that it should be considered an essential process, which should run in parallel to ensure that the foundation for the PAEC assessment is progressively improved over time.

In conjunction with the development of the PAEC protocol and the first assessment of arctic tundra ecosystems, statistical models have been developed which address ecological state variables/indicators subjected to multiple and interacting drivers. These have been targeted particularly at indicators with associated phenomena of relatively low validity. These have served to quantify the relative importance of drivers (Marolla et al. 2021), separate the effects of manageable and non-manageable drivers (Henden et al. 2020; Nater et al. 2021), and strengthen the understanding of the role of indicators in the ecosystem (Ims et al. 2019; Mellard et al. 2022). These models further provide an adaptive framework for continuous updates as new monitoring data are added, and in some cases a basis for providing near-term forecasts (Henden et al. 2020; Marolla et al. 2021), which is an essential step forward in adaptive management.

While rigorous, model-based attributions of change in indicators to specific drivers may be hindered by lack of data or inadequate knowledge basis, the most challenging tasks in PAEC regard the higher levels in the assessment hierarchy (characteristics and ecosystem level), and especially when it comes to accounting for diverse sources of uncertainty. For instance, what is an *adequate indicator coverage* for complex ecosystem characteristics is not straightforward to ascertain. Indeed, most of the uncertainty sources in ecosystem state assessments cannot be assessed in quantitative terms (Supplementary Table S3). Moreover, for uncertainty components that need to be assessed qualitatively there are no unambiguous criteria for setting the borders between nominal levels, e.g., between the levels *adequate* and *partially adequate* for indicator coverage or between *intermediate* or *low* validity for phenomena. For the assessment of the condition of the whole ecosystem, it is required that the panel can integrate all sources of uncertainty when assessing the overall evidence. As there exist no rigorous formula for doing this, the final narrative ecosystem level assessment needs to be expressed verbally in a way that shows how the significant sources of uncertainty identified in the hierarchy (Supplementary Table S3) are considered.

### Future integration of stakeholders in PAEC

An assessment according to PAEC is primarily a scientific exercise. However, PAEC is also envisioned to be a tool for adaptive management of ecosystems, or specific ecosystem components (see below). Lessons learned from global assessments, including recent reviews of the IPBES, demonstrate that the ingredients behind a successful ‘knowledge-to-impact-and-action’ transfer is close engagement, commitment and dialog across the science-policy interface (Krug et al. 2020; Ruckelshaus et al. 2020; Stevance et al. 2020; Press 2021). Thus, the PAEC protocol allows for the integration of a stakeholder group, consisting for instance of representatives for management agencies responsible for the particular ecosystem, into the assessment process. This is non-mandatory, but such co-production of assessments across the science-policy interface will serve to increase policy relevance (Balvanera et al. 2020; Jackson 2021; Martin et al. 2023). Policy impact will also increase by broadening PAEC, from a purely scientific assessment with post hoc engagement with stakeholders, to an operational platform for enhancing informed decision-making regarding adaptive management strategies and the implementation and evaluation of specific management actions. While the PAEC protocol sketches one possible approach for stakeholder involvement (Jepsen et al. 2020), many possible approaches exist from a relatively peripheral involvement to a true integration in all levels of the assessment following a more formalized framework for stakeholder involvement in structured planning and decision-making (Armitage et al. 2011; Cook et al. 2014; Solomonsz et al. 2021). Irrespectively of the approach agreed upon for stakeholder involvement, it must follow a structured and transparent prior agreement as to the exact role of the stakeholder group versus the scientific panel in each phase of the assessment, the types of stakeholders which can be involved, and the types of input they can provide.

### PAEC as a platform for adaptive and knowledge driven management

By focusing on expected (phenomena) and observed change in trajectories (time series analyzes), PAEC is coherent with the paradigm of studying and managing rates of ecological change (Williams et al. 2021). In a climate change perspective, the globe’s ecosystems are inevitably destined to change. Hence, aiming at managing rates rather than states appears to be the most rational strategy for ecosystem-based management (Williams et al. 2021; Crausbay et al. 2022; Magness et al. 2022). In this regard, definitions of reference states (e.g., for climate) could just serve as a means for assessing deviations - as they are used in PAEC – and not as management goals. Accordingly, Barnosky et al. (2017) suggested that, rather than attempting to hold ecosystems to an idealized conception of the past as has been the prevailing management paradigm, maintaining vibrant ecosystems for the future requires new approaches. Focusing on change trajectories, facilitates adaptive and open-ended management strategies when the outcomes and endpoints of transformative ecosystem change are unknown (Jackson 2021), due to non-equilibrium (non-stationary) dynamics (Carpenter and Brock 2006; Turner et al. 2020), and the large uncertainty of the extents and impacts of future global change (Schindler and Hilborn 2015). Modifying change trajectories (i.e. slowing down or altering direction) by means of ecosystem-level interventions may be possible whenever the ecosystem is subjected to manageable drivers of change, e.g., land-use and harvest (Prober et al. 2019; Lynch et al. 2022). It should also be noted that by including a broad knowledge base about expected responses to drivers in the phenomena, PAEC may help identify lagged responses likely to occur in the future, thus giving time to prepare for management of change trajectories.

PAEC offers opportunities to assess the significance of change rates along a continuum from abrupt to slow (Williams et al. 2021), and according to their ecological significance and certainty; i.e., in terms of scientific validity and evidence of the phenomena. However, as many of the phenomena represent ecosystem services, ecosystem-based management also involves considerations and priorities that are beyond the realm of natural sciences. Indeed, a tight interaction between ecosystem scientists, managers and policy makers is needed to tackle the huge challenge of achieving sustainable management of ecosystems subjected to multiple stressors including climate change (Williams et al. 2020). As discussed above, PAEC can provide a platform for such interactions. Indeed, structured stakeholder involvement has already been probed successfully in case of the Norwegian Low Arctic ecosystem (Hamel et al. 2022). Moreover, the holistic ecosystem-based management plans that have been established for many marine areas, typically involve interactions with stakeholders as a key step in the implementation process (O’Boyle and Jamieson 2006; Curtin and Prellezo 2010; Long et al. 2015; Röckmann et al. 2015), and are prime candidates for putting this principle into practical use.

Assessing cumulative impacts, and the complexity of ecosystem responses to compound driver effects, is a challenging scientific task. For PAEC it requires contributions from panel members that have expert knowledge about the specifics of separate indicators and phenomena, as well as members that have competence to make a holistic assessment of how the whole set of indicators/phenomena represents ecosystem condition. The latter, which in our experience is most demanding, must ultimately be based on a theoretical framework, i.e., some ecosystem model. Ecosystem models range from simple verbal descriptions of links between a few state variables to complex end-to-end mathematical representations of ecosystem structures and processes (Geary et al. 2020). Although end-to-end ecosystems models may be the most advanced analytical approach, such models are not available for most ecosystems. Moreover, end-to-end ecosystem models are most useful for strategic purposes (Geary et al. 2020), while not for empirical assessments of ecosystem condition. Different theoretical frameworks may be needed depending on which are the key processes and change drivers in the focal ecosystem. The applications of the PAEC protocol to the Norwegian Arctic tundra ecosystems were guided by a set of conceptual food web models, which helped identifying the links between phenomena and ecosystem characteristics needed for making the ecosystem-level assessment. Importantly the same conceptual models guided the ecosystem-based monitoring systems that generated much of the baseline data for the PAEC assessment (Ims et al. 2013, Ims and Yoccoz 2017). Indeed, PAEC may serve as an essential integral component of adaptive monitoring and management of rapidly transforming ecosystems.

## Supplementary information

Supplementary Information
