# Supplementary material for: Panel-based assessment of ecosystem condition as a platform for adaptive and knowledge driven management
Source: Environ Manage. 2024 Sep 13;74(5):1020–36. doi: 10.1007/s00267-024-02042-9 (PMC11438735; doi:10.1007/s00267-024-02042-9)
Supplement: Supplementary file 1 — Supplementary Information [file 267_2024_2042_MOESM1_ESM.pdf]

Supplementary information

Panel-based assessment of ecosystem condition as a platform for adaptive and knowledge driven management

Jane U. Jepsen[1*], Per Arneberg[2], Rolf A. Ims[3], Anna Siwertsson[2,5], Nigel G. Yoccoz[3], Per Fauchald[1], Åshild Ø. Pedersen[4], Gro I. van der Meeren[2], Cecilie H. von Quillfeldt[4]

[1]Norwegian Institute for Nature Research, Department for Arctic Ecology, Fram Centre, 9296 Tromsø, Norway

[2] Institute of Marine Research, Department of Ecosystem Processes, Fram Centre, 9296 Tromsø, Norway

[3] UiT The Arctic University of Norway, Department of Arctic and Marine Biology, 9037 Tromsø, Norway.

[4] Norwegian Polar Institute, 9296 Tromsø, Norway

[5]Current address: Akvaplan-niva, Fram Centre, 9296 Tromsø, Norway

*Corresponding author: jane.jepsen@nina.no

S1. Ecosystem characteristics

Table S1. The seven ecosystem characteristics used in the Norwegian assessments of ecosystem condition (Nybø and Evju 2017).

Ecosystem characteristic	Role
Primary productivity	The total production of live plant biomass is a fundamental function that impinges on most other functions and structures of the ecosystem
Biomass distribution among trophic levels	How biomass is distributed among trophic levels is a structural characteristic that reflects trophic interactions and the flow of energy and matter in food webs
Functional groups within trophic levels	The abundance of groups of species with similar traits (e.g., growth forms, guilds), highlights functional aspects of community structure
Functionally important species and biophysical structures	Individual species (or those physical structures they represent) that maintain functions which are essential to the functioning of the ecosystem
Landscape ecological patterns	Spatial features, such as habitat patch size and configuration, both reflect and determine ecological processes and anthropogenic disturbances
Biological diversity	The change (turnover) in genetic or species composition, especially declines or loss of species that characterize the focal ecosystem (e.g., endemics) in its reference state
Abiotic factors	Chemical and physical state variables within an ecosystem that force and interact with biological processes and structures

S2. Case study: Assessment of the Norwegian part of the North Sea shelf ecosystem

The application of PAEC to the Norwegian part of the North Sea, following up a national action plan for biodiversity, was part of a larger undertaking of assessing ecosystem condition for all marine and terrestrial ecosystem types in Norway. The case study includes results from the first operational assessment of this region, conducted by a scientific panel consisting of 24 scientists in 2021-2023 (Arneberg et al. 2023).

Delineation of the Norwegian North Sea shelf ecosystem and history of anthropogenic impact

The Norwegian part of the North Sea shelf ecosystem is located in the northern part of the North Sea and Skagerrak. The spatial extent of the assessed ecosystem is based on the Norwegian management plan for the North Sea and Skagerrak. It includes areas outside the baseline, in Norway's territorial waters and exclusive economic zone northwards to latitude 62 °N. (Fig. S2.1). The shelf ecosystem is further limited to areas shallower than 200 m depth. The North Sea has a long history of anthropogenic impact, including depletion or extirpation of large whale populations in historical times (ICES 2021). Industrial fisheries date back to at least the 19th century and have caused large transformations of the fish communities (Greenstreet et al. 1999; Jennings and Blanchard 2004; Thurstan et al. 2010; Sodeland et al. 2022). Anthropogenic input of nutrients, largely from rivers, and to a smaller extent from the atmosphere, led to eutrophication in the North Sea from the first half of the 20th century, affecting coastal regions most seriously (Ærtebjerg et al. 2001). The problem has ceased after an agreement in 1988 to reduce nutrient input to the North Sea (OSPAR 2017). In the most recent decades, increasing impact from climate change has been seen, affecting a large range of ecological groups and processes, including important zooplankton groups with consequences for fish stocks through recruitment failures (e.g., Beaugrand and Kirby 2010).

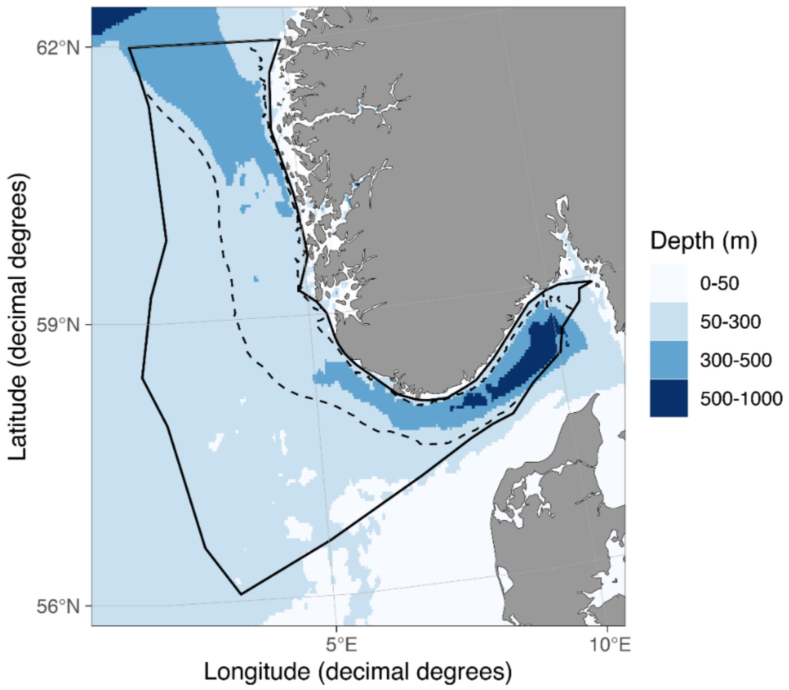

Fig. S2.1. Delineation of the Norwegian North Sea shelf ecosystem. Note that the water column and sea floor deeper than 200 meters in the Norwegian Trench (indicated by the dotted line) is defined as a separate ecosystem (Nybø and Evju 2017) and not included in the current assessment, while the water column above 200 meters in these areas is included.

Reference condition

The reference condition was defined in the Norwegian *System for assessment of ecological condition* (Nybø and Evju 2017) as "intact ecosystems" for all marine and terrestrial ecosystems. This reference condition implies that fundamental structures, functions, and productivity of the ecosystem are maintained, and that these are not significantly impacted by modern industrial human influences, including anthropogenic climate change. For more details about the reference condition, see the main text of the paper under the case study of the Norwegian Low Arctic Tundra.

Assessments of individual ecosystem characteristics

Below we summarize the outcome of the assessment for the seven ecosystem characteristics and the ecosystem as a whole. For the full assessment, including assessment of the knowledge base and individual indicators and supporting references, we refer to Arneberg et al. (2023).

Abiotic factors

The assessment of this ecosystem characteristic is based on seven indicators: *Temperature, Stratification, Flow conditions, Nutrients, Light attenuation, pH* and *Aragonite saturation* (Fig. S2.3). Combined these give a good representation of aspects of the abiotic system that are affected by anthropogenic drivers and those that mediate this impact to the biological parts of the ecosystem (Payne et al. 2009; Beaugrand and Kirby 2010; Holt et al. 2018; Opdal et al. 2019; Gao et al. 2021). Thus, indicator coverage is assessed as *adequate* for this ecosystem characteristic (Fig. S2.3).

The phenomena for these indicators include several well studied driver links from climate research, such as increasing temperature caused by greenhouse gas emissions and other anthropogenic impact on climate (IPCC 2019). As there is also good evidence of the climate related responses for the biological parts of the system (Payne et al. 2009; Beaugrand and Kirby 2010; Lindemann and St John 2014), most of these phenomena are assessed to have high validity. Exceptions are phenomena related to ocean acidification, which have intermediate validity due to fragmented understanding of effects on ecosystems (Browman 2016) (Fig. S2.2). *Temperature* [NP19] was assessed as having a strong weight a priori to the assessment of the ecosystem characteristic because of its importance as a proxy of other physical properties and as a driver for the ecosystem structure and functioning.

Based on analyses of time series observations, the phenomenon for *Temperature* [NP39] shows high evidence for change, reflecting a substantial increase in temperature within a 70-year long time series (Fig. S2.4a). *Light attenuation*, *pH* and *Aragonite saturation* [NP43], [NP44], [NP45] show limited evidence for change, while remaining phenomena show no evidence. Based on the assessed importance of an increase in temperature, the scientific panel nevertheless concluded that there is *substantial deviation* from the reference condition for the characteristic *Abiotic factors* (Fig. S2.3). The uncertainty related to this conclusion is mainly related to short time series for some phenomena (Table S2).

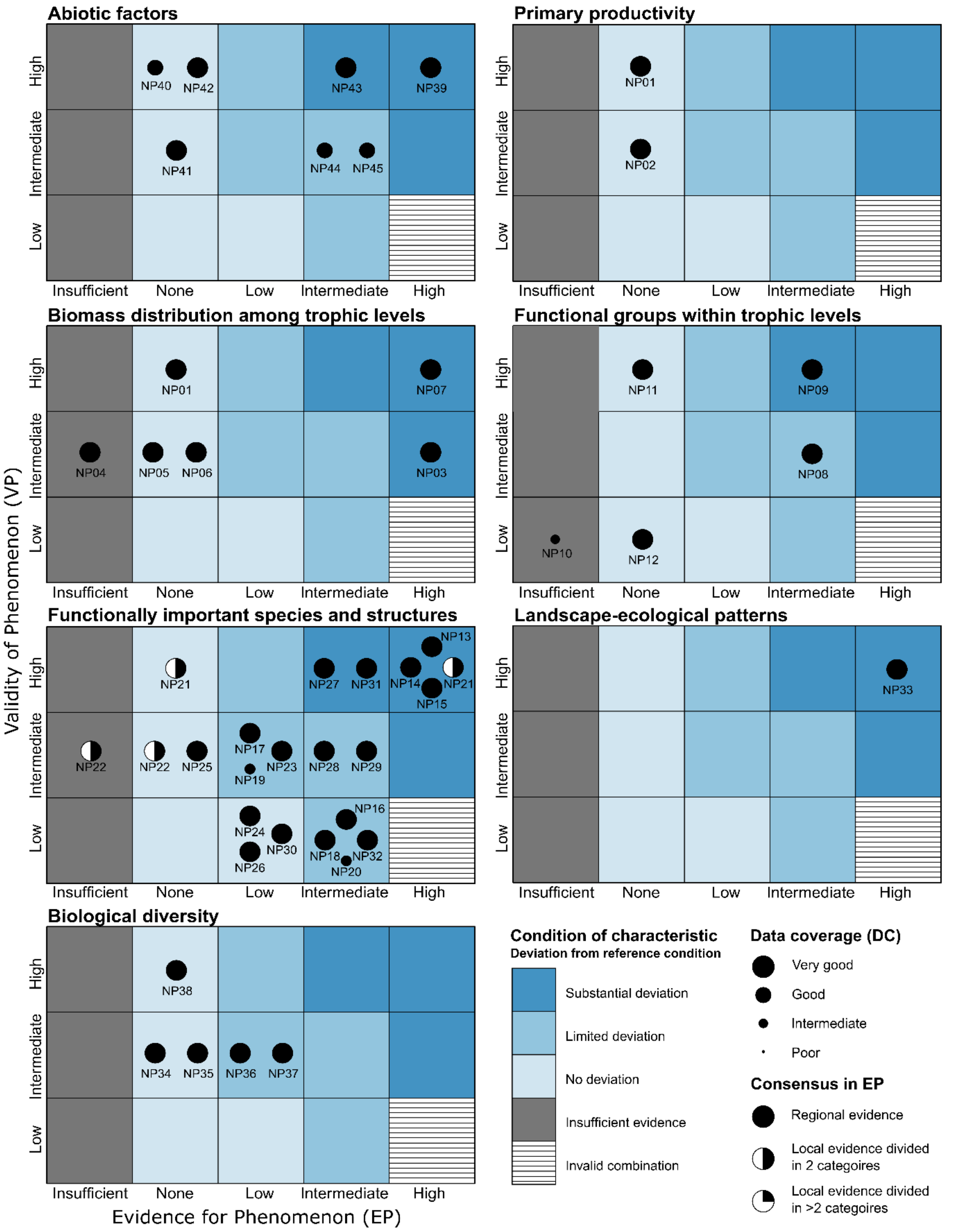

Fig. S2.2. PAEC assessment diagrams for the case study of the Norwegian North Sea, used to guide the assessment of the seven ecosystem characteristics and the ecosystem as a whole. The diagrams show how all phenomena are placed along the axis of validity (VP) and evidence (EP). Data coverage can be taken into account by placing lower weight on phenomena represented with smaller symbols. Lack of consensus in the level of evidence (EP), for instance due to contrasting trends in different regions or data sources, is indicated by splitting the symbols for EP into half or quarters. Legend for phenomena numbers and timespan for available data are shown in Table S2.

Table S2. Phenomena included in the case study of the Norwegian North Sea, and timespan for the associated time-series data used in trend analyses.

ID	Phenomena	Time-series
NP01	Increasing annual primary productivity	2003-2020
NP02	Change in the spring bloom timing	2003-2020
NP03	Decreasing abundance of herbivorous copepods	1958-2016
NP04	Increasing abundance of carnivorous zooplankton	1958-2016
NP05	Change biomass of low trophic level fish	2000-2021
NP06	Decreasing biomass of high trophic level fish	2000-2021
NP07	Decline in abundance of high trophic level seabirds	1970/1980-2020
NP08	Changes in Meroplankton vs. Holoplankton composition	1958-2016
NP09	Reduced average copepod community body size	1958-2016
NP10	Increasing biomasses of gelatinous zooplankton	2000-2021
NP11	Decreasing fish community mean body size	2000-2021
NP12	Decreasing proportion of slow-life species and increasing proportion of fast life species	2000-2021
NP13	Decrease in abundance of *C. finmarchicus* relative to abundance of *C. helgolandicus*	1958-2016
NP14	Declining abundance of *Pseudocalanus* spp. and *Paracalanus* spp.	1958-2016
NP15	Decreasing cod stock size	1963-2022
NP16	Decreasing cod recruitment	1963-2022
NP17	Decreasing haddock stock size	1972-2022
NP18	Decreasing haddock recruitment	1972-2022
NP19	Decreasing saithe stock size	1967-2022
NP20	Decreasing saithe recruitment	1967-2022
NP21	Decreasing lesser sandeel stock size	1986-2022
NP22	Decreasing lesser sandeel recruitment	1986-2022
NP23	Stable Norway pout stock size	1983-2022
NP24	Stable Norway pout recruitment	1983-2022
NP25	Decreasing whiting stock size	1978-2022
NP26	Decreasing whiting recruitment	1978-2022
NP27	Decreasing herring stock size	1947-2022
NP28	Decreasing herring recruitment	1947-2022
NP29	Decreasing mackerel stock size	1980-2021
NP30	Change in mackerel recruitment	1980-2021
NP31	Decreasing shrimp stock size	1907-2021
NP32	Decreasing shrimp recruitment	1971-2021
NP33	Decreasing area unimpacted by bottom trawling	2017-2020
NP34	Decreasing biomass of fish vulnerable to higher temperatures	2000-2021
NP35	Increasing biomass of fish benefitting from higher temperatures	2000-2021
NP36	Decrease in number of copepod species sensitive to higher temperatures	1958-2016
NP37	Increase in number of “warm-water" copepod species	1958-2016
NP38	Decreasing biomass of fish species vulnerable to fisheries	2000-2021
NP39	Warming of the water column	1952-2021
NP40	Increasing stratification of the upper water column	2005-2021
NP41	Increasing inflow of Atlantic water to the North Sea	1985-2021
NP42	Increasing concentration of nutrients	1980-2021
NP43	Increase in light attenuation	1903-1998
NP44	Decreasing pH	2012-2020
NP45	Decreasing aragonite saturation	2012-2020

Primary productivity

Two indicators form the basis for the assessment of this ecosystem characteristic: *Annual primary productivity* and *Timing of the spring bloom* (Fig. S2.3). These two indicators are linked to several key processes in marine ecosystems, such as production at higher trophic levels and temporal overlap with zooplankton reproduction (Edwards and Richardson 2004; Chassot et al. 2010; Durant et al. 2019). The indicator coverage is considered as *partly adequate*, mainly due to the lack of indicators related to phytoplankton species composition and *in situ* measurements of primary productivity (Fig. S2.3).

The phenomenon associated with the indicator for *Annual primary productivity* [NP01] is assessed to have high validity (Fig. S2.2), as the knowledge about the link to the drivers (climate change and anthropogenic input of nutrients (Lindemann and St John 2014; OSPAR 2017; Holt et al. 2018)) is assessed as certain and there is robust evidence for substantial ecosystem consequences of changes in annual primary productivity (Chassot et al. 2010). For *Timing of the spring bloom* [NP02], the phenomenon is assessed as having intermediate validity (Fig. S2.2), as although the consequences of changes are considered to be well understood, climate change can have two opposing effects on spring bloom timing: a delaying effect acting through water darkening caused by increased terrestrial runoff and a push for earlier blooms caused by increased water column stratification in a warmer climate (Racault et al. 2012; Opdal et al. 2019).

Analyses of observation time series show no evidence of change in *Annual primary productivity* (Fig. S2.4b) or *Timing of the spring bloom*. Thus, the scientific panel concluded that there is *no deviation* from the reference condition for the characteristic *Primary productivity* (Fig. S2.3), but that there is large uncertainty associated with this conclusion, as the time series for both phenomena are short (Table S2) and do not capture periods of strong trends in climate change, the main driver.

Biomass distribution among trophic levels

Six indicators were considered for the assessment of this ecosystem characteristic, covering primary producers, two groups of zooplankton (herbivorous and carnivorous, respectively), two groups of fish (low and high trophic level, respectively) and high trophic level seabirds (Fig. S2.3). For one of the indicators (*Carnivorous zooplankton*), the data were classified as insufficient, because the group is not considered to be sampled appropriately by the platform used, the Continuous Plankton Recorded Survey (CPR) (Richardson et al. 2006). The assessment was thus based on five indicators, which cover the most important biomass pools of the ecosystem. Still, indicators on microbes and groups representing a considerable part of the biomass above secondary consumers, such as benthos and marine mammals are lacking. Given these gaps, the indicator coverage for this ecosystem characteristic is rated as partially adequate (Fig. S2.3).

Validity is assessed as high for the phenomena associated with the indicators *Annual primary productivity* (see assessment of the ecosystem characteristic "Primary productivity" above) and *High trophic level seabirds*. The latter constitute a significant part of the top predator guild in the ecosystem and there is substantial evidence of the link to fisheries (Moland et al. 2013; Grémillet et al. 2018; Norderhaug et al. 2021). For *Herbivorous copepods* and *Low trophic level fish*, a good understanding of ecosystem consequences from change in indicator values (Beaugrand et al. 2003; Fauchald et al. 2011), but less certain understanding of links to drivers contribute to intermediate validity of the associated phenomena. For *High trophic level fish*, intermediate validity is contributed by a good understanding of the link to the main driver (fisheries) (Jennings et al. 2002) and less good understanding of ecosystem consequences (Fig. S2.2).

Analyses of time series observations show marked declines for *Herbivorous copepods* [NP03] and *High trophic level seabirds* [NP07], for the latter near extirpation of many fish-eating species in the region. The evidence for change is thus assessed as high for the associated phenomena. For the three other indicators, *Annual primary productivity, Low trophic level fish* and *High trophic level fish,* no evidence

of change is evident from the time series observations (Fig. S2.2). Based on this, the scientific panel concluded that the ecosystem characteristic *Biomass distribution among trophic levels* exhibit limited deviation from the reference condition (Fig. S2.3), the core argument being that while several indicators show no expected change, some do, representing development towards an impacted state that may require attention. This conclusion comes with a high degree of uncertainty, because the time series for the indicators showing no change are short and does not cover major changes in the drivers (climate change and fisheries) (Table S2). Thus, if the time series had stretched back to before the warming that started in the 1980s and before periods of substantial overfishing that was addressed by the implementation of the new EU fisheries policy in 2003, more extensive changes may have been observed.

<u>Functional groups within trophic levels</u>

Five indicators were available for the assessment of this ecosystem characteristic. One of these (*Biomass of gelatinous zooplankton*) was not included as the data quality was considered insufficient (due to an inappropriate sampling protocol and a short time series) (Fig. S2.3). Of the four indicators that the assessment was based on, two address variation in ecological functions among fish; *Fish body size* may reflect variation in the many functions that are related to body mass (Fisher et al. 2010; Andersen et al. 2016) and *Fish life history* address variation in occurrence of fast-life vs. slow-life species, which is generally linked to population resilience and several other ecological functions (Winemiller 2005). In addition, two indicators reflecting variation in ecological functions in the zooplankton community is included: *Copepod body size,* which may reflect variation in energy-transfer efficiency to higher trophic levels, a key aspect of ecosystem functioning (Lewandowska and Sommer 2010), and *Holoplankton vs. Meroplankton,* which may capture variation in interspecific competition experienced from meroplanktonic larvae by copepods, as well as variation in bentho-pelagic coupling (Kirby et al. 2007; Kirby et al. 2008). Relevant indicators for functions performed by phytoplankton, microbes, parasites, seabirds and mammals are lacking, and the indicator coverage for this ecosystem characteristic is therefore rated as *partially adequate* (Fig. S2.3).

For *Copepod body size* and *Fish body size* the understanding of the link to the main drivers (climate change and fisheries, respectively) is rated as certain and the understanding of ecosystem consequences from change in the indicators rated as good. The associated phenomena are consequently assessed as having high validity. The phenomenon associated with *Holoplankton vs. Meroplankton* is assessed as having intermediate validity due to a good understanding of the link to the main driver (climate change, acting mainly on reproduction of a single important species, the sea urchin *Echinocardium cordatum*; Kirby et al. (2007)), but a less good understanding of ecosystem consequences from change. For *Fish life history* understanding of the link to main drivers and ecosystem role is rated as less good and the associated phenomenon assessed as having low validity (Fig. S2.2).

For *Copepod body size* [NP09]*,* time series analyses show a marked decline in average species body size, but evidence for change is still assessed as intermediate because changes within species are not included. A similar assessment is given for *Holoplankton vs. Meroplankton* [NP08], where a marked increase in the relative abundance of meroplankton is seen (mainly due to an increase of *E. cordatum* larvae). For the two fish indicators, no change is seen for the period covered by the available time series (Fig. S2.2, Table S2). Based on this evidence, the scientific panel assessed this ecosystem characteristic as showing evidence for *limited deviation* from the reference condition, where the changes in copepod body size and abundance of meroplankton vs. holoplankton indicate a more impacted condition that may require attention (Fig. S2.3). The main uncertainty around this choice of assessment category is linked to the short length of the time series for the fish indicators, which means that they do not overlap with periods of change in the main drivers (climate change and fisheries) (Table S2).

Functionally important species and biophysical structures

The assessment of this ecosystem characteristic is based on 20 indicators. The majority (16) are related to stock sizes and recruitment of important fish species, but also indicators for shrimp and important zooplankton groups are included (Fig. S2.3). These cover a large fraction of what is considered the functionally most important species. However, indicators are lacking for sharks and whales and microbial species, where the latter could have provided information about the microbial loop. Indicator coverage is therefore assessed as *partially adequate* (Fig. S2.3).

The validity of the phenomena for these indicators varies from high to low (Fig. S2.2). Phenomena with high validity are associated with six indicators for stock size of well-studied species; cod, herring, sandeel, shrimp and copepod species of the genera *Calanus, Pseudocalanus and Paracalanus*, for which there is a good understanding of links to drivers and ecosystem consequences of changes in indicator values (e.g., Payne et al. 2009; Beaugrand and Kirby 2010; Dickey-Collas et al. 2010; Montero et al. 2021). The phenomena with low validity are all associated with seven indicators for recruitment, for which links to drivers are inherently difficult to understand (e.g., Haltuch et al. 2019; Garcia et al. 2021). Intermediate validity is seen for seven phenomena associated with some recruitment indicators and indicators for stock size for less well studied species, such as whiting and Norway pout. The phenomena for copepods [NP13], [NP14] and cod [NP15] and herring stocks [NP27] were assigned higher importance than other phenomena prior to the assessment as these species were considered to have particularly important roles for the dynamics of the ecosystem, for example through the effects that changes in copepod communities can have on fish stocks (Payne et al. 2009; Beaugrand and Kirby 2010).

Evidence for expected change is assessed as high or intermediate for 11 of the 20 phenomena for this ecosystem characteristic and split between none and high for different geographic regions for yet another phenomenon (Fig. S2.2). In addition, the four phenomena that had been assigned a higher weight than others are all placed in the category *substantial deviation* (Fig. S2.2). Examples of two of these indicators (*Pseudo- and Paracalanus*, and *Cod stock size*), which both show profound declines during a long time period, are shown in Figs. S2.4c,d. The scientific panel therefore concluded that the ecosystem characteristic *Functionally important species and biophysical structures* exhibit *substantial deviation* from the reference condition (Fig. S2.3). There is little uncertainty about this choice of assessment category, considering the number of indicators showing the same signal of impact on the ecosystem characteristic condition. For several of the phenomena, time series are long enough to capture major changes in the drivers (Table S2), in particular climate change and fisheries, and this contributes to the low level of uncertainty in the assessment. Phenomena with lower evidence also have lower validity and it is likely that improving their validity through research would further decrease the uncertainty of the assessment. In addition, if indicators for species not included, such as sharks or whales, had been added, these would likely have supported the current assessment of substantial deviation from the reference conditions, as there is evidence that these groups were significantly more abundant in historical times (ICES 2021).

Landscape-ecological patterns

This ecosystem characteristic is assessed based on a single indicator, *Area unimpacted by bottom trawling*. This indicator is covering the major landscape-shaping driver in the North Sea but could be completed by an estimation of area occupied by human infrastructures, noise or light habitats for marine species, or areas invaded by marine species. The indicator coverage for this ecosystem characteristic is therefore rated as *partially adequate* (Fig. S2.3).

Knowledge of ecosystem consequences from change in the indicator is rated as certain, as multiple studies have shown how bottom trawling may reduce the complexity of seabed habitats and affect the functioning and productivity of benthic ecosystems (Hiddink et al. 2011; van Denderen et al. 2013;

Buhl-Mortensen et al. 2016). As the link to the driver is obvious, the validity of the associated phenomenon is thus assessed as high (Fig. S2.2).

Based on an indicator that estimates degree of impact from trawling on benthic communities (Pitcher et al. 2022) and extent of trawling in the assessment area, there is evidence that a significant part of the assessment area is impacted by bottom trawling to a degree that is likely to have considerable ecosystem consequences. The evidence for the phenomenon is thus assessed as high (Fig. S2.2). Based on this, the scientific panel concluded that this ecosystem characteristic is showing evidence for *substantial deviation* from the reference condition (Fig. S2.3). There is little uncertainty associated with this conclusion, as although other indicators could be added (e.g. on area of noise habitat), abounding literature are showing that these new indicators would support it.

<u>Biological diversity</u>

Five indicators form the basis for assessment of this ecosystem characteristic (Fig. S2.3). All of them aim at measuring change that can be attributed to particular drivers., Two indicators for zooplankton are *Copepod species vulnerable to higher temperature* and *Copepod species benefitting from higher temperature*. For fish two similar indicators are *Fish species vulnerable to higher temperature* and *Fish species benefitting from higher temperature* and an additional indicator for *Fish species vulnerable to fisheries*. While this covers important groups in the ecosystem, indicators for microbial species, parasites, phytoplankton, benthos, seabirds and marine mammals are lacking, and indicator coverage for this ecosystem characteristic is rated as *partially adequate* (Fig. S2.3).

The validity of the phenomena associated with these indicators range from intermediate to high. For the fish indicators, knowledge about the link to drivers (climate change or fisheries) is rated as certain, and knowledge about ecosystem role as good for *Fish species vulnerable to fisheries* and less good for the other two fish indicators, giving high validity for the phenomenon associated with the former indicator and intermediate validity for the two latter. For the zooplankton indicators, validity of the associated phenomena are assessed as intermediate, as knowledge about the link to climate change is rated as less certain and knowledge about ecosystem role as good based on studies that have indicated links to recruitment in fish and seabirds (Beaugrand et al. 2003; Wanless et al. 2005; Beaugrand et al. 2009) (Fig. S2.2).

Based on long time series (Table S2), some evidence for expected change is observed for the zooplankton indicators [NP36], [NP37], and evidence for the associated phenomena is assessed as low. For the fish indicators, no evidence of change expected under increasing human impact is seen, but they are based on short time series not covering the period of change in the drivers (Fig. S2.2, Table S2). Based on this evidence, the scientific panel concluded that the ecosystem characteristic *Biological diversity* is showing evidence for *limited deviation* from the reference condition, with the tendencies for declines in cold-water and increase in warm-water copepods representing warning signals that biological diversity in the North Sea may require attention (Fig. S2.3). Uncertainties associated with this assessment is mainly related to short time series for the fish indicators and lack of indicators for other groups than zooplankton and fish.

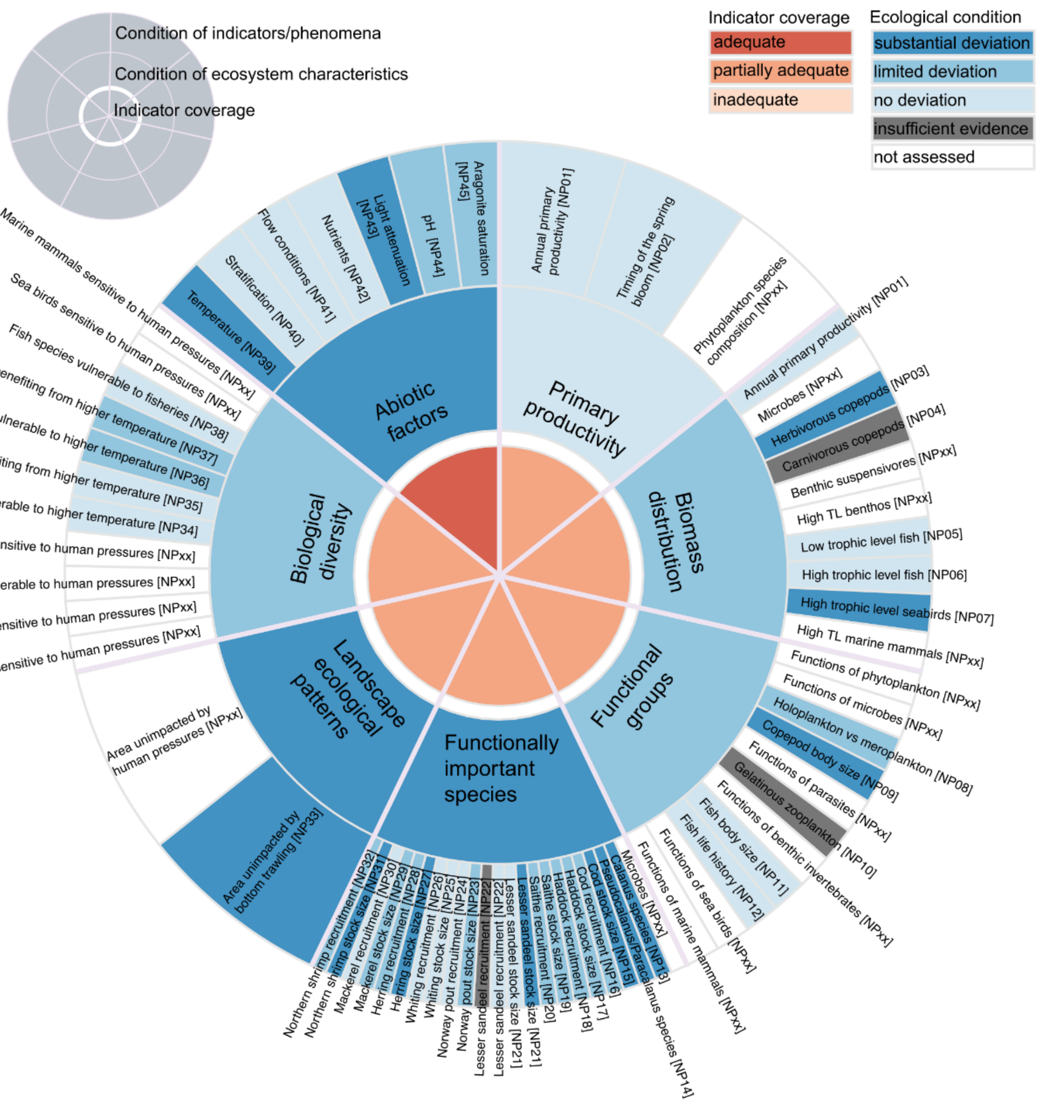

Fig. S2.3. A graphical summary of the assessment of ecological condition for the Norwegian North Sea shelf ecosystem. The outer ring shows the assessment of condition at the level of individual indicators with associated phenomena IDs in square brackets. Indicators that the scientific panel has recommended for inclusion, but which have not yet been included, are shown in white to illustrate the perceived most important deficiencies in the indicator data set. The middle ring shows the assessment of condition at the level of ecosystem characteristics, and the innermost ring shows the quality of the indicator coverage.

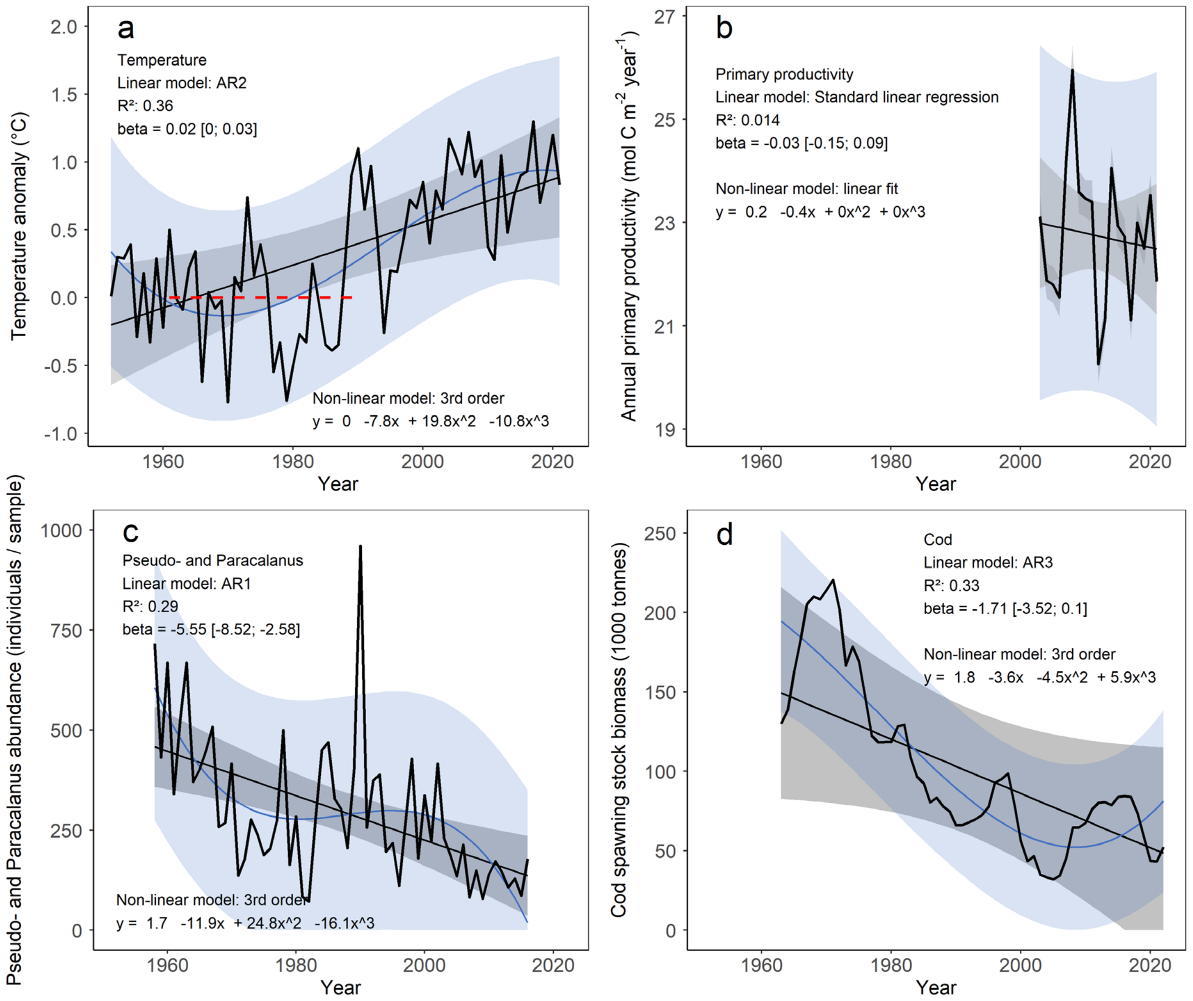

Fig. S2.4. Selected time series of indicators used in the assessment for the ecosystem characteristics *Abiotic factors* (a), *Primary productivity* (b) and *Functionally important species and biophysical structures* (c, d) for the Norwegian North Sea shelf ecosystem. Note that the length of available time series varies, and for some biological indicators (shown for *Annual primary productivity* here) do not overlap with the chosen baseline for climate (1961-1990).

Details on (a), Temperature [NP39]: The black fluctuating line shows the temperature anomalies relative to the reference period for climate (1961-1990), indicated by the red stippled line. Monthly or quarterly temperature observations were taken from 100-200 m depth along two transects in the North Sea (Torungen-Hirtshals, and Utsira-Orkney), and values represent annual means. The black regression line shows the rate of change (±2SE), and the blue curve shows the best fitted non-linear model (95% prediction interval).

Details on (b), Annual primary productivity [NP01]: The black fluctuating line shows the mean (±SE) of annual primary productivity, based on satellite observations. The black regression line shows the rate of change (±2SE). The linear trend was selected as the best fitting model when allowing for non-linearity (95% prediction interval in blue).

Details on (c), *Pseudocalanus* and *Paracalanus* species [NP14]: The fluctuating black line shows the annual mean (March – September) abundance of *Pseudo-* and *Paracalanus* species based on observations from the Continuous Plankton Recorder Survey. The black regression line shows the rate of change (±2SE), and the blue curve shows the best fitted non-linear model (95% prediction interval).

Details on (d), Cod stock size [NP15]: The black fluctuating line shows the estimated spawning stock biomass of cod in the entire North Sea, Skagerrak, and the eastern English Channel (ICES 2022). The black regression line shows the rate of change (±2SE), and the blue curve shows the best fitted non-linear model (95% prediction interval).

For time series longer than 50 years (a, c, d), regression models with different structures for the residuals were used to estimate linear rates of change. The best fitting model was chosen based on Akaike Information Criterion (AIC). The possible models included were: 1) AR0, a standard linear regression with independent residuals, 2) AR1, a 1st order autoregressive model, 3) AR2, a 2nd order autoregressive model, 4) AR3, a 3rd order autoregressive model. In addition, potential non-linear trends were identified using the first step of a TREC analysis (Solvang and Planque 2020; Solvang and Ohishi 2022). Different polynomial models, from 1st to 3rd degree, were fitted to standardized time series and the best fitting model was selected based on the AIC criterion. For time series shorter than 50 years (b), standard linear regression was fitted only to indicate the direction of change. Possible non-linear trends were explored using the TREC approach.

Overall ecosystem condition

The conclusion about the condition of the overall ecosystem is summarised in a single paragraph that is aimed at wide communication to management, stakeholders, the scientific community and the public. For the current assessment, this reads as follows:

> "The scientific panel concludes that the ecosystem in the Norwegian sector of the North Sea and Skagerrak is substantially impacted by human pressures. There is little uncertainty about this conclusion. Climate change is strongly affecting abiotic conditions, mainly through increasing temperatures. This is substantially impacting the rest of the ecosystem, along with other drivers, in particular fisheries. Consequences are largest for functionally important species and seafloor habitats. There are also signs of human-driven changes in diversity of species and ecological functions, as well as in the trophic pyramid of the ecosystem. With further climate change and development of industrial activities in the North Sea, human pressures will remain intensive in the future."

A more extensive summary of the changes observed, how they may be linked and how they have contributed to the assessment is as follows:

The indicators describing the ecosystem characteristic *Abiotic factors* deviates substantially from the reference condition. This is due to a change in temperature linked to anthropogenic climate change and that have large implications for the rest of the ecosystem. There has also been a significant darkening of the water, which is related to climate change through increased runoff of organic material from land to sea and which may affect several processes in the North Sea and Skagerrak, such as timing of the spring bloom and interactions between predators and prey. Despite short time series (9 years), signs of increased ocean acidification (reduced pH and aragonite saturation) are detected, but there are large uncertainties about the biological consequences of this. Concentrations of nutrients have previously been elevated but have declined during the last decades because of improved management of runoff from agriculture and other terrestrial sources.

The main rise in temperature occurred at the end of the 1980s, as a marked warming of about 1°C above the long-term mean, over a few years. An important biological response was a change in the zooplankton species that form the main link between the primary producing phytoplankton and higher trophic levels in the ecosystem. During the "cold" state of the ecosystem, the zooplankton species *Calanus finmarchicus* dominated. This species hibernates during winter and generates a large production peak when it spawns in spring. Following the warming, there has been a shift in dominance towards *Calanus helgolandicus*, which feeds throughout the year, spawns in summer and autumn and does not produce marked seasonal peaks in production. The shift in temperature has also caused a general decline in *Paracalanus* and *Pseudocalanus* zooplankton species. Together, these changes in the zooplankton community have had large consequences for the amount and type of food, and the time of the year it is available for higher trophic levels.

The two zooplankton indicators for copepod species described above (*Calanus* species and *Paracalanus*/*Pseudocalanus* species) are important contributors to the substantial deviation from the

reference condition observed for the ecosystem characteristic *Functionally important species and biophysical structures*. For fish, a contributor to the changes observed is overfishing in the 1980s and 1990s that brought several stocks to low levels. After overfishing was reversed following the implementation of the new EU fishery policy from 2003, recovery of several of the stocks has been hampered by low recruitment in the 2000s and 2010s. The zooplankton groups described above are important food for fish larvae. For cod and herring, which are well studied species, the recruitment failures have been clearly linked to the changes in the zooplankton community driven by the warming. For other fish species, which have been less studied, there are larger uncertainties about the causes of recruitment failures, but given similarities in biology with herring and cod, an influence of the warming-driven changes in the zooplankton community described above is a relevant hypothesis. For shrimp, a recruitment failure which took place in the 2000s caused the stock to collapse under a fishing pressure that was no longer sustainable. Thus, for this species, the recruitment failure preceded the stock decline. Sandeel in the northern part of the North Sea (Vikingbanken) has not recovered from previous overfishing and is at a low level, while sandeel in the southern part of the Norwegian sector of the North Sea has recovered.

Another important aspect of human impact on the ecosystem is the large fraction of the seabed and benthic communities affected by bottom trawling, prompting the ecosystem characteristic *Landscape-ecological patterns* to be assessed as deviating substantially from the reference condition. This may affect complexity of seabed habitats and functioning and productivity of benthic ecosystems, which are aspects of the ecosystem that there are no direct indicators for in the assessment.

For the ecosystem characteristic *Distribution of biomass among trophic levels*, which reflects the overall shape of the trophic pyramid of the ecosystem, it is assessed that there is evidence for limited changes away from the reference condition. This is due to declines in abundance of herbivorous copepods (reflecting to a large extent the same climate-driven changes that are described for *Calanus*, *Paracalanus* and *Pseudocalanus* species above) and fish-eating seabirds likely caused by a combined effect of fisheries and climate change. While the declines in seabird populations are dramatic, seabirds still constitute a small part of the overall biomass in the ecosystem under the reference condition, and the major impact on the shape of the trophic pyramid therefore stems from the changes in herbivorous copepod abundance. There are uncertainties associated with the assessment because time series are short for the lowest (primary production) and intermediate (fish) levels of the trophic pyramid and are lacking for benthos (intermediate trophic level) and marine mammals (top trophic level). It is likely that fish and marine mammals were present in higher abundance under the reference conditions. It is however noted that climate driven changes in fish biomass (indicated for many functionally important fish species above), may be compensated by influx of southern species, thus conserving the relative contribution of fish in the trophic pyramid.

The changes observed in the ecosystem characteristic *Functional groups within trophic levels*, which again, are due to changes in the zooplankton community observed with long time series, show that the important copepod part of this community has changed towards smaller-sized species. This is expected to have significant effects on the predators of the copepods, which includes larva and adults of many fish species, and potentially propagate to other parts of the ecosystem. The marked increase in the abundance of plankton from species that are planktonic only in parts of the life cycle (typically larvae, such as for many benthic species) compared with abundance of species that are planktonic through the whole life cycle (e.g., copepods and other species referred to as zooplankton in this text) contributes further to the changes, and the short time series for fish indicators (for which no change is seen) introduces uncertainty to the assessment.

For the ecosystem characteristic *Biological diversity*, it was assessed that there is evidence for limited anthropogenic impact. Based on long time series, moderate changes linked to climate change are seen for zooplankton species, with a tendency for species sensitive to increases in temperatures to occur less frequently and species expected to benefit from this occurring more frequently. For groups of fish,

no changes in species occurrence were seen, but this was assessed with considerably shorter time series not covering changes in climate and fisheries. This introduces uncertainty to the assessment.

Finally, no change was seen for the two indicators contributing to the assessment of the ecosystem characteristic *Primary productivity* (yearly production and timing of the spring bloom). Again, the time series for these indicators are short and not covering change in the main drivers (temperature, light regime, and nutrients), meaning we do not know whether there has indeed been change due to impact from human activities.

S3. Sources of uncertainty in ecosystem condition assessment

Table S3. Sources of uncertainty in ecosystem condition assessment. Uncertainty ranges from linguistic uncertainty linking ecological concepts and hypotheses to actual measurements (Houle et al. 2011), to epistemic uncertainty associated with natural variation, stochasticity, sampling and modelling (Regan et al. 2002). The structured process of PAEC aims at a systematic analysis of these different contributions to overall uncertainty. This table describes the main types and sources of uncertainty, how they are approached in the PAEC protocol, and draw on examples from both presented cases (i.e., Low Arctic tundra, **LAT**, and the Norwegian North Sea, **NNS**; the latter presented in Supplementary Material S2).

**PHASE: step**	**Potential sources of uncertainty**	**Approach in PAEC**	**Examples from LAT and NNS cases**
SCOPING: Delineation of ecosystem extent	Linguistic:<br>Multiple definitions of the same ecosystem can exist, based on abiotic, biotic or combined criteria.<br><br>Epistemic:<br>Natural variation in abiotic conditions. Uncertainties in map sources, remote sensing data, or predictive models.	Sources of uncertainty related to ecosystem delineation described in text only.	**LAT**: Delineated by the Barents Sea coast to the north and by the approximate forest line to the south. Roughly corresponding to the climatic extent of the CAVM bioclimatic sub-zones D and E. Uncertain delineation relative to northern alpine tundra.<br>**NNS**: Horizontal extent based on the Norwegian Management Plan area. The shelf ecosystem defined as shallower than 200 m depth. Uncertain ecological relevance of administrative borders and uncertainty about interactions with the ecosystem in deeper waters.
SCOPING: Definition of the reference condition	Linguistic:<br>Definitions may rely on highly ambiguous concepts such as “intact” or “pristine” which can only be described in normative terms.<br><br>Epistemic:<br>Natural variation in abiotic conditions underlying climatic references. Limited or missing sampling coverage for historical reference periods. Uncertainties in map sources, remote sensing data, or predictive models.	Generic with respect to reference condition. If the definition of the reference condition is unambiguous, it is described in quantitative terms, if not in normative terms only.<br>Phenomena are formulated relative to the reference condition.	**LAT/NNS:** The reference condition is ambiguously defined as an «intact» state. It is described in normative terms only for the ecosystem as a whole, each characteristic and each indicator. The climatic baseline is unambiguously defined as the 1961-1990 normal and is quantified (mean and variation) for climate-derived indicators.

PHASE: step	Potential sources of uncertainty	Approach in PAEC	Examples from LAT and NNS cases
SCOPING:<br>Choice and definitions of ecosystem characteristics	Linguistic:<br>Definitions of ecosystem characteristics can be ambiguous (e.g., what is included in the concepts l*andscape-ecological patterns* or *biological diversity*?).<br><br>Epistemic:<br>Ecosystem characteristics are related/overlapping.	The role of each characteristic towards the final assessment, including how it relates to other characteristics, is described in text only.	**LAT/NNS:** The set of characteristics used in the presented cases was mandated.<br>The UN SEEA ecosystem condition typology (United Nations et al. 2021) uses a broadly overlapping set of characteristics – abiotic (physical and chemical), biotic (composition: e.g., diversity, structure: e.g., biomass and vegetation coverage, function: e.g., primary productivity and disturbance frequency), and landscape (connectivity, fragmentation)
SCOPING:<br>Choice of indicators	Linguistic:<br>Ambiguous definitions of ecosystem characteristics (see above) propagate to the choice of indicators.<br>Diverging practices regarding the components of, or metrics used to represent, given indicators.<br><br>Epistemic:<br>Uncertain or unknown representativity of indicators. Robustness and understanding of the relationship between state variables and surrogate indicators (Lindenmayer et al. 2015).	Each ecosystem characteristic is represented by multiple indicators. How well the total indicator set covers the different facets of a given characteristic is expressed by the *Indicator coverage (IC).* Qualitative assessment to three categories (Fig. S2.3).	**LAT/NNS:** The ecosystem characteristics *Primary productivity* is represented by indicators relating to maximum productivity, timing of spring greening (**LAT**) and timing of spring bloom (**NNS**). **LAT** is supplemented by a local field-based indicator on vegetation biomass of key vegetation strata. High reliance on remote sensing data sources, and few in-situ measurements, contributes to uncertainty both regarding cause (attribution) and effect (which species/species groups are responsible for observed changes). Indicator coverage for this characteristic is hence assessed as only *partially adequate* for both ecosystems.
SCOPING:<br>Spatial, temporal representativity of available data	Linguistic/Epistemic:<br>Uncertain delineation of ecosystem extent (see above) will propagate uncertainties in the assessment of spatial representativity.<br><br>Epistemic:<br>The extent to which data are based on probability sampling (with strata defined to improve precision) or model-based sampling focusing on estimating causal relationships (Buckland et al. 2000; Albert et al. 2010). The extent to which temporal coverage allows for	*Spatial representativity (SR)*: Evaluated relative to the chosen ecosystem extent (target population), depending on whether each data source is based on probability or model-based sampling. Qualitative assessment to four categories.<br><br>*Temporal representativity (TR):*<br>Evaluated relative to the natural dynamics of the indicator and the extent to which relevant seasonality has been	**NNS**: Estimation of saithe recruitment is done using data from design-based monitoring that does not cover coastal regions. In addition, seasonality is important but not covered, and although the resulting time series is long enough for representing relevant dynamics, reference conditions are not covered. Data coverage is therefore set to "intermediate", the second lowest category.

PHASE: step	Potential sources of uncertainty	Approach in PAEC	Examples from LAT and NNS cases
	separating natural variation of ecosystem indicators from the effects of drivers. Will depend on the importance of fast versus slow dynamics in the focal ecosystem.	accounted for in the sampling. Qualitative assessment to four categories.<br><br>Overall *Data coverage (DC)* is based on the scores for SR and TR. Qualitative assessment to four categories (Fig. S2.2).	
SCOPING: Formulation and validity of phenomena	<u>Linguistic:</u><br>Formulation of phenomena in words, not equations with unambiguous state variables. What is defined as ecologically significant?<br><br><u>Epistemic:</u><br>Choice of relevant literature, assessment of levels of evidence, quantification of ecological change	The scientific support for phenomena is assessed based on scientific literature with respect to:<br>i) The level of certainty between relevant drivers and change in a given indicator. Qualitative assessment to two categories.<br>ii) The level of understanding of the implication of change (ecological significance) for ecosystem condition. Qualitative assessment to two categories.<br><br>Based on the choice of categories above, *the Validity of phenomena (VP)* is assessed. Qualitative assessment to three categories (Fig. S2.2).	**NNS:** Climate change is considered as the main driver for the indicator *Timing of the spring bloom*. Two effects leading to opposing trends are described. One is acting through increased terrestrial runoff (because of increased precipitation caused by climate warming), leading to increased darkening of the water and a delay of the spring bloom. Another acts through warming of the water, causing increased stratification of the water column, leading to an earlier bloom. Thus, the expected trend is simply formulated as "Change in the spring bloom timing", and the knowledge about the link between the indicator and the driver is assessed as "less certain". Knowledge about consequences for the ecosystem from changes in spring bloom timing is rated as "good", and based on this, the score for the *Validity of the phenomenon (VP)* rated as 'intermediate'.
ANALYSIS: Trend analysis	<u>Epistemic:</u><br>Model specification will determine how trustworthy is inference about reference condition, existing trends, and attribution to drivers. It can include measurement error of drivers and response, which pathways are included, linearity of relationships, statistical distributions, and independence.	Observed changes in indicators are analyzed to assess the statistical evidence for the hypothesized changes described in the phenomenon. This can take the form of time-series analysis (is there a change in the expected direction and magnitude) or preferably dynamic causal models accounting for direct and indirect impacts (e.g., climate and management), i.e., attribution analysis.	**LAT:** Selected time series with trend and uncertainty estimates are shown in Fig. 3. These show the trend in the indicator *Snow cover duration* (*Abiotic factors*), contrasting trends in the indicator *Maximum vegetation productivity* (*Primary productivity*), and contrasting trends in voles and lemmings within the *rodent community* (*Functionally important species and biophysical structures*).<br>**NSS**: Selected time series with trend and uncertainty estimates are shown in Fig. S2.4. These show the linear and non-linear trends in the indicator *Temperature*

PHASE: step	Potential sources of uncertainty	Approach in PAEC	Examples from LAT and NNS cases
			(*Abiotic factors*), *Annual primary productivity* (*Primary productivity*), and *Pseudocalanus and Paracalanus species* and *Cod* (*Functionally important species and biophysical structures*).
ASSESSMENT: Evidence for phenomenon	<u>Linguistic:</u><br>Limits between qualitative categories (vs. grey shading or continuous grading of evidence in IPCC (Mastrandrea et al. 2011), or the confidence framework of IPBES (IPBES 2018)).<br><br><u>Epistemic:</u><br>Estimated effect size and its statistical uncertainty. The estimated effect size and its spatial and temporal consistency need to be compared to other studies (see SCOPING – phenomenon) and statistical uncertainty can be assessed using categories, e.g., IPCC virtually certain = 99-100% probability (Mastrandrea et al. 2011).	The *Evidence for phenomena (EP)* depends on the estimated effect size (quantitative component), as well as the importance of the indicator and characteristic for the condition of the ecosystem (qualitative component).<br><br>The resulting *EP* is a qualitative assessment to five categories (Fig. S2.2), following practices from IPBES and IPCC that such assessments do not use a probabilistic certainty scale.	**NNS**: The indicator *Pseudocalanus and Paracalanus* (Fig. S2.4C) shows strong statistical evidence for the expected decrease described in the phenomenon. The magnitude of change is large (roughly a halving of the index over 70 years) and expected ecosystem significance is high. *EP* is hence rated to the highest category.
ASSESSMENT: Deviation from reference condition - Ecosystem characteristics level	<u>Linguistic:</u><br>Limits between qualitative categories (no change, limited and substantial change) that correspond to a gradient of changes.<br><br><u>Linguistic/Epistemic:</u><br>Propagating uncertainties and combining qualitative categories describing degrees of evidence, validity, and consistency.	A textual assessment and a qualitative assessment to three categories based on the placement of each phenomenon along the axis of *Validity of phenomena (VP)* and *Evidence for phenomena (EP)* (Fig. 4, see Fig. S2.2 for definitions of categories).<br>It synthesizes the evidence, validity, and consistency of changes across all phenomena within an ecosystem characteristic, as well as the main sources of uncertainty in the choice of assessment category.	**LAT:** The ecosystem characteristic *Abiotic factors* is assessed as having *substantial deviation* from the reference condition (e.g., the highest category). It is based on 11 indicators, of which 8, related to temperature and snow, are in the upper left part of the assessment diagram (high *VP*, high *EP*). The remaining, related to precipitation and basal ice, show no or limited change. The assessment panel argues that the latter are partly of less ecological relevance, and partly based on data with high uncertainty (gridded precipitation data based on few stations) and conclude that the assessment category for this characteristic can be chosen with a high level of confidence.
ASSESSMENT: Deviation from	<u>Linguistic:</u>	A textual assessment which synthesizes the nature and extent of changes	**LAT/NNS:** Both ecosystems showed a mixture from no deviation to substantial deviation at the characteristic

**PHASE: step**	**Potential sources of uncertainty**	**Approach in PAEC**	**Examples from LAT and NNS cases**
reference condition - Ecosystem level	Limits between qualitative categories (no change, limited and substantial deviation).<br><br><u>Linguistic/Epistemic:</u><br>Propagating uncertainties and combining qualitative categories of deviations at the characteristic level to an ecosystem level.	observed across all characteristics, the main sources of uncertainty in the overall conclusions, the current state of knowledge of the reference condition (baseline for change), and the main drivers of observed changes. The hierarchy induced by the choice of characteristics needs to be accounted for in the final assessment. It should not induce a kind of a priori weighting, rather the importance of each characteristic should be based on its ecological importance and its relationship to other characteristics.	level. This can be due to either lagged effects, caused by for example slow response of tundra vegetation to climate change, or lack of empirical evidence for changes, for example linked to poor indicator or data coverage. For **LAT**, the deviation at an ecosystem level was assessed as limited as most biotic characteristics were not yet significantly affected by the main driver, climate change. For **NNS**, the deviation at an ecosystem level was assessed as substantial, with strong impacts from climate change and other drivers, particularly fisheries. A robust knowledge base and long time series for key phenomena, which covers long term changes in the most important drivers, are important contributors to the low level of uncertainty.
ASSESSMENT: Knowledge gaps and future trajectories	<u>Linguistic/Epistemic:</u><br>Knowledge gaps should relay the main sources of uncertainties identified in the whole assessment. Uncertainties for future trajectories depend on uncertainties regarding internal ecosystem dynamics interacting with uncertainties regarding drivers (e.g., management).	PAEC encourages the use of general conceptual models of ecosystem structure and functions as a basis for identifying and prioritizing knowledge gaps. Highest priority is placed on identifying the knowledge gaps contributing most to uncertainty in the overall assessment (e.g., improving inadequate *indicator coverage (IC)*, *data coverage (DC)*, and phenomena validity through improved understanding of causal relationships between drivers and indicators/state variables).<br>Future trajectories are briefly discussed with focus on how the ecosystem likely changes in the near future, given the observed changes in drivers and indicators.	**LAT:** Knowledge gaps contributing to uncertainty in the assessment were identified at all levels in the hierarchical assessment. Examples are i) decomposition is not considered, and should be included as a new ecosystem characteristic, or, as a minimum, as one or more indicators under existing characteristics (decomposers as functional groups), ii) indicator coverage is less than adequate for six of seven characteristics, and seven specific new indicators are recommended for development to improve this, iii) inadequate understanding of complex multi-driver-response relationships should be addressed using more advanced model-based analysis to improve *validity (VP)* and confidence in attribution.

References

Albert, C. H., N. G. Yoccoz, T. C. Edwards Jr, C. H. Graham, N. E. Zimmermann, and W. Thuiller. 2010. Sampling in ecology and evolution – bridging the gap between theory and practice. Ecography **33**:1028-1037. https://doi.org/10.1111/j.1600-0587.2010.06421.x

Andersen, K. H., T. Berge, R. J. Gonçalves, M. Hartvig, J. Heuschele, S. Hylander, N. S. Jacobsen, C. Lindemann, E. A. Martens, A. B. Neuheimer, K. Olsson, A. Palacz, A. E. F. Prowe, J. Sainmont, S. J. Traving, A. W. Visser, N. Wadhwa, and T. Kiorboe. 2016. Characteristic sizes of life in the oceans, from bacteria to whales. Pages 217-+ *in* C. A. Carlson and S. J. Giovannoni, editors. Annual Review of Marine Science, Vol 8. 10.1146/annurev-marine-122414-034144

Arneberg, P., B. Husson, A. Siwertsson, J. Albretsen, K. Børsheim, C. Denechaud, J. Durant, T. Falkenhaug, P. Fauchald, A. Opdal, S. Jentoft, T. Johannessen, E. Johnsen, E. Jones, C. Kvamme, G. Ljungström, P. Mortensen, Y. Reecht, H. Solvang, M. Skogen, A. Slotte, E. Strand, G. Søvik, and G. van der Meeren. 2023. Panel-based Assessment of ecosystem condition of the North Sea shelf ecosystem. Rapport fra Havforskningen nr. 2023-17.

Beaugrand, G., K. M. Brander, J. A. Lindley, S. Souissi, and P. C. Reid. 2003. Plankton effect on cod recruitment in the North Sea. Nature **426**:661-664. 10.1038/nature02164

Beaugrand, G., and R. R. Kirby. 2010. Climate, plankton and cod. Global Change Biology **16**:1268-1280. 10.1111/j.1365-2486.2009.02063.x

Beaugrand, G., C. Luczak, and M. Edwards. 2009. Rapid biogeographical plankton shifts in the North Atlantic Ocean. Global Change Biology **15**:1790-1803. 10.1111/j.1365-2486.2009.01848.x

Browman, H. I. 2016. Applying organized scepticism to ocean acidification research Introduction. ICES Journal of Marine Science **73**:529-536. 10.1093/icesjms/fsw010

Buckland, S. T., I. B. Goudie, and D. L. Borchers. 2000. Wildlife population assessment: past developments and future directions. Biometrics **56**:1-12. 10.1111/j.0006-341x.2000.00001.x

Buhl-Mortensen, L., K. E. Ellingsen, P. Buhl-Mortensen, K. L. Skaar, and G. Gonzalez-Mirelis. 2016. Trawling disturbance on megabenthos and sediment in the Barents Sea: chronic effects on density, diversity, and composition. ICES Journal of Marine Science **73**:98-114. 10.1093/icesjms/fsv200

Chassot, E., S. Bonhommeau, N. K. Dulvy, F. Mélin, R. Watson, D. Gascuel, and O. Le Pape. 2010. Global marine primary production constrains fisheries catches. Ecology Letters **13**:495-505. https://doi.org/10.1111/j.1461-0248.2010.01443.x

Dickey-Collas, M., R. D. M. Nash, T. Brunel, C. J. G. van Damme, C. T. Marshall, M. R. Payne, A. Corten, A. J. Geffen, M. A. Peck, E. M. C. Hatfield, N. T. Hintzen, K. Enberg, L. T. Kell, and E. J. Simmonds. 2010. Lessons learned from stock collapse and recovery of North Sea herring: a review. ICES Journal of Marine Science **67**:1875-1886. 10.1093/icesjms/fsq033

Durant, J. M., J. C. Molinero, G. Ottersen, G. Reygondeau, L. C. Stige, and O. Langangen. 2019. Contrasting effects of rising temperatures on trophic interactions in marine ecosystems. Scientific Reports **9**. 10.1038/s41598-019-51607-w

Edwards, M., and A. J. Richardson. 2004. Impact of climate change on marine pelagic phenology and trophic mismatch. Nature **430**:881-884. 10.1038/nature02808

Fauchald, P., H. Skov, M. Skern-Mauritzen, D. Johns, and T. Tveraa. 2011. Wasp-waist interactions in the North Sea ecosystem. PLOS ONE **6**. https://doi.org/10.1371/journal.pone.0022729

Fisher, J. A. D., K. T. Frank, and W. C. Leggett. 2010. Global variation in marine fish body size and its role in biodiversity-ecosystem functioning. Marine Ecology Progress Series **405**:1-13. 10.3354/meps08601

Gao, S., S. S. Hjollo, T. Falkenhaug, E. Strand, M. Edwards, and M. D. Skogen. 2021. Overwintering distribution, inflow patterns and sustainability of Calanus finmarchicus in the North Sea. Progress in Oceanography **194**. 10.1016/j.pocean.2021.102567

Garcia, T., B. Planque, P. Arneberg, B. Bogstad, O. Skagseth, and M. Tiedemann. 2021. An appraisal of the drivers of Norwegian spring-spawning herring (Clupea harengus) recruitment. Fisheries Oceanography **30**:159-173. 10.1111/fog.12510

Greenstreet, S. P. R., F. E. Spence, and J. A. McMillan. 1999. Fishing effects in northeast Atlantic shelf seas: patterns in fishing effort, diversity and community structure. V. Changes in structure of the North Sea groundfish species assemblage between 1925 and 1996. Fisheries Research **40**:153-183. 10.1016/s0165-7836(98)00210-0

Grémillet, D., A. Ponchon, M. Paleczny, M. L. D. Palomares, V. Karpouzi, and D. Pauly. 2018. Persisting worldwide seabird-fishery competition despite seabird community decline. Current Biology **28**:4009-+. 10.1016/j.cub.2018.10.051

Haltuch, M. A., E. N. Brooks, J. Brodziak, J. A. Devine, K. F. Johnson, N. Klibansky, R. D. M. Nash, M. R. Payne, K. W. Shertzer, S. Subbey, and B. K. Wells. 2019. Unraveling the recruitment problem: A review of environmentally-informed forecasting and management strategy evaluation. Fisheries Research **217**:198-216. 10.1016/j.fishres.2018.12.016

Hiddink, J. G., A. F. Johnson, R. Kingham, and H. Hinz. 2011. Could our fisheries be more productive? Indirect negative effects of bottom trawl fisheries on fish condition. Journal of Applied Ecology **48**:1441-1449. 10.1111/j.1365-2664.2011.02036.x

Holt, J., J. Polton, J. Huthnance, S. Wakelin, E. O'Dea, J. Harle, A. Yool, Y. Artioli, J. Blackford, J. Siddorn, and M. Inall. 2018. Climate-driven change in the North Atlantic and Arctic oceans can greatly reduce the circulation of the North Sea. Geophysical Research Letters **45**:11827-11836. 10.1029/2018gl078878

Houle, D., C. Pélabon, Günter P. Wagner, and Thomas F. Hansen. 2011. Measurement and Meaning in Biology. The Quarterly Review of Biology **86**:3-34. 10.1086/658408

ICES. 2021. Greater North Sea ecoregion – Ecosystem overview. In Report of the ICES Advisory Committee, ICES Advice 2021, Section 9.1 **ICES Advice 2021, Section 9.1**. https://doi.org/10.17895/ices.advice.9434

ICES. 2022. Cod (*Gadus morhua*) in Subarea 4, Division 7.d, and Subdivision 20 (North Sea, eastern English Channel, Skagerrak). . Report of the ICES Advisory Committee, 2022. ICES Advice 2022, cod.27.47d20.

IPBES. 2018. IPBES Guide on the production of assessments. Secretariat of the Intergovernmental Science-Policy Platform on Biodiversity and Ecosystem Services, Bonn, Germany

IPCC. 2019. IPCC Special Report on the Ocean and Cryosphere in a Changing Climate. Cambridge University Press, Cambridge, UK and New York, NY, USA. https://doi.org/10.1017/9781009157964.

Jennings, S., and J. L. Blanchard. 2004. Fish abundance with no fishing: predictions based on macroecological theory. Journal of Animal Ecology **73**:632-642. 10.1111/j.0021-8790.2004.00839.x

Jennings, S., S. P. R. Greenstreet, L. Hill, G. J. Piet, J. K. Pinnegar, and K. J. Warr. 2002. Long-term trends in the trophic structure of the North Sea fish community: evidence from stable-isotope analysis, size-spectra and community metrics. Marine Biology **141**:1085-1097. 10.1007/s00227-002-0905-7

Kirby, R. R., G. Beaugrand, and J. A. Lindley. 2008. Climate-induced effects on the meroplankton and the benthic-pelagic ecology of the North Sea. Limnology and Oceanography **53**:1805-1815. 10.4319/lo.2008.53.5.1805

Kirby, R. R., G. Beaugrand, J. A. Lindley, A. J. Richardson, M. Edwards, and P. C. Reid. 2007. Climate effects and benthic-pelagic coupling in the North Sea. Marine Ecology Progress Series **330**:31-38. 10.3354/meps330031

Lewandowska, A., and U. Sommer. 2010. Climate change and the spring bloom: a mesocosm study on the influence of light and temperature on phytoplankton and mesozooplankton. Marine Ecology Progress Series **405**:101-111. 10.3354/meps08520

Lindemann, C., and M. A. St John. 2014. A seasonal diary of phytoplankton in the North Atlantic. Frontiers in Marine Science **1**. 10.3389/fmars.2014.00037

Lindenmayer, D., J. Pierson, P. Barton, M. Beger, C. Branquinho, A. Calhoun, T. Caro, H. Greig, J. Gross, J. Heino, M. Hunter, P. Lane, C. Longo, K. Martin, W. H. McDowell, C. Mellin, H. Salo, A. Tulloch, and M. Westgate. 2015. A new framework for selecting environmental surrogates. Science of the Total Environment **538**:1029-1038. https://doi.org/10.1016/j.scitotenv.2015.08.056

Mastrandrea, M. D., K. J. Mach, G. K. Plattner, O. Edenhofer, T. F. Stocker, C. B. Field, K. L. Ebi, and P. R. Matschoss. 2011. The IPCC AR5 guidance note on consistent treatment of uncertainties: a common approach across the working groups. Climatic Change **108**:675-691. https://doi.org/10.1007/s10584-011-0178-6

Moland, E., E. M. Olsen, H. Knutsen, P. Garrigou, S. H. Espeland, A. R. Kleiven, C. André, and J. A. Knutsen. 2013. Lobster and cod benefit from small-scale northern marine protected areas: inference from an empirical before - after control-impact study. Proceedings of the Royal Society B-Biological Sciences **280**. 10.1098/rspb.2012.2679

Montero, J. T., M. Lima, S. A. Estay, and E. L. Rezende. 2021. Spatial and temporal shift in the factors affecting the population dynamics of Calanus copepods in the North Sea. Global Change Biology **27**:576-586. 10.1111/gcb.15394

Norderhaug, K. M., K. Nedreaas, M. Huserbråten, and E. Moland. 2021. Depletion of coastal predatory fish sub-stocks coincided with the largest sea urchin grazing event observed in the NE Atlantic. AMBIO **50**:163-173. 10.1007/s13280-020-01362-4

Nybø, S., and M. Evju, editors. 2017. Fagsystem for fastsetting av god økologisk tilstand. Forslag fra et ekspertråd. Ekspertrådet for økologisk tilstand, Trondheim, Norway.

Opdal, A. F., C. Lindemann, and D. L. Aksnes. 2019. Centennial decline in North Sea water clarity causes strong delay in phytoplankton bloom timing. Global Change Biology **25**:3946-3953. https://doi.org/10.1111/gcb.14810

OSPAR. 2017. Third Integrated Report on the Eutrophication Status of the OSPAR Maritime Area. OSPAR report **694/2017**:165pp.

Payne, M. R., E. M. C. Hatfield, M. Dickey-Collas, T. Falkenhaug, A. Gallego, J. Groger, P. Licandro, M. Llope, P. Munk, C. Rockmann, J. O. Schmidt, and R. D. M. Nash. 2009. Recruitment in a changing environment: the 2000s North Sea herring recruitment failure. ICES Journal of Marine Science **66**:272-277. https://doi.org/10.1093/icesjms/fsn211

Pitcher, C. R., J. G. Hiddink, S. Jennings, J. Collie, A. M. Parma, R. Amoroso, T. Mazor, M. Sciberras, R. A. McConnaughey, A. D. Rijnsdorp, M. J. Kaiser, P. Suuronen, and R. Hilborn. 2022. Trawl impacts on the relative status of biotic communities of seabed sedimentary habitats in 24 regions worldwide. Proceedings of the National Academy of Sciences of the United States of America **119**. 10.1073/pnas.2109449119

Racault, M. F., C. Le Quere, E. Buitenhuis, S. Sathyendranath, and T. Platt. 2012. Phytoplankton phenology in the global ocean. Ecological Indicators **14**:152-163. https://doi.org/10.1016/j.ecolind.2011.07.010

Regan, H. M., M. Colyvan, and M. A. Burgman. 2002. A taxonomy and treatment of uncertainty for ecology and conservation biology. Ecological Applications **12**:618-628. https://doi.org/10.1890/1051-0761(2002)012[0618:ATATOU]2.0.CO;2

Richardson, A. J., A. W. Walne, A. W. G. John, T. D. Jonas, J. A. Lindley, D. W. Sims, D. Stevens, and M. Witt. 2006. Using continuous plankton recorder data. Progress in Oceanography **68**:27-74. 10.1016/j.pocean.2005.09.011

Sodeland, M., S. Jentoft, P. E. Jorde, M. Mattingsdal, J. Albretsen, A. R. Kleiven, A. E. W. Synnes, S. H. Espeland, E. M. Olsen, C. Andre, N. C. Stenseth, and H. Knutsen. 2022. Stabilizing selection on Atlantic cod supergenes through a millennium of extensive exploitation. Proceedings of the National Academy of Sciences of the United States of America **119**. https://doi.org/10.1073/pnas.2114904119

Solvang, H., and M. Ohishi. 2022. trec: An R package for trend estimation and classification to support integrated assessment of the marine ecosystem and environmental factors. arXiv:2209.06619. . https://doi.org/10.48550/arXiv.2209.06619.

Solvang, H. K., and B. Planque. 2020. Estimation and classification of temporal trends to support integrated ecosystem assessment. ICES Journal of Marine Science **77**:2529-2540. https://doi.org/10.1093/icesjms/fsaa111

Thurstan, R. H., S. Brockington, and C. M. Roberts. 2010. The effects of 118 years of industrial fishing on UK bottom trawl fisheries. Nature Communications **1**. https://doi.org/10.1038/ncomms1013

United Nations et al. 2021. System of Environmental-Economic Accounting—Ecosystem Accounting (SEEA EA). White cover publication, pre-edited text subject to official editing.

van Denderen, P. D., T. van Kooten, and A. D. Rijnsdorp. 2013. When does fishing lead to more fish? Community consequences of bottom trawl fisheries in demersal food webs. Proceedings of the Royal Society B-Biological Sciences **280**. 10.1098/rspb.2013.1883

Wanless, S., M. P. Harris, P. Redman, and J. R. Speakman. 2005. Low energy values of fish as a probable cause of a major seabird breeding failure in the North Sea. Marine Ecology Progress Series **294**:1-8. 10.3354/meps294001

Winemiller, K. O. 2005. Life history strategies, population regulation, and implications for fisheries management. Canadian Journal of Fisheries and Aquatic Sciences **62**:872-885. 10.1139/f05-040

Ærtebjerg, G., J. Carstensen, K. Dahl, J. Hansen, K. Nygaard, B. Rygg, K. Sørensen, G. Severinsen, S. Casartelli, W. Schrimpf, C. Schiller, and J. N. Druon. 2001. Eutrophication in Europe's coastal waters. 7/2001, Topic report, European Environment Agency.
